# Supplementary material for: Breaking the photoswitch speed limit
Source: Nat Commun. 2023 Nov 20;14:7556. doi: 10.1038/s41467-023-43405-w (PMC10660956; doi:10.1038/s41467-023-43405-w)
Supplement: Supplementary file 1 — Supplementary Information [file 41467_2023_43405_MOESM1_ESM.pdf]

# Supplementary Information
## Breaking the Photoswitch Speed Limit
Thaggard et al.

**Table of Contents:**


1. Supplementary Note 1 6
2. Supplementary Methods 6
3. Supplementary Note 2 11
4. Supplementary Note 3 13
5. **Supplementary Table 1**. XYZ coordinates of **2** in which the structures of the –COOH groups were fixed 13
6. **Supplementary Table 2**. XYZ coordinates of **3** in which the structures of the pyridine groups were fixed 14
7. **Supplementary Table 3**. Geometric parameters and excitation energies for coordinatively-integrated and non-coordinatively-integrated **3** 14
8. **Supplementary Table 4**. Calculated energies of non-coordinatively-integrated **2** as a function of $r^1_{C–O}$ and $r^2_{C–O}$ 15
9. **Supplementary Table 5**. Calculated energies of coordinatively-integrated **2** as a function of $r^1_{C–O}$ and $r^2_{C–O}$ 17
10. **Supplementary Table 6**. Estimated parameters for non-coordinatively-integrated and coordinatively-integrated **2** 19
11. **Supplementary Table 7**. Calculated energies at the saddle point of non-coordinatively-integrated and coordinatively-integrated **2** 19
12. **Supplementary Table 8**. Geometric parameters and calculated excitation energies for non-coordinatively-integrated and coordinatively-integrated **2** 19
13. **Supplementary Table 9**. X-ray crystal structure refinement data for **2** 20
14. **Supplementary Table 10**. Comparison of literature and experimental photoisomerization rate constants 21
15. **Supplementary Figure 1**. Synthetic route for preparation of 1',3',3'-trimethyl-6-nitrospiro[chromene-2,2'-indoline]-5'-carboxylic acid (**2**) 22
16. **Supplementary Figure 2**. Synthetic route for preparation of 4-(2-(1-(4-carboxyphenyl)-2-ethoxy-2-oxoethylidene)hydrazineyl)benzoic acid (**6**) 22
17. **Supplementary Figure 3**. Bond lengths and dihedral angle of **2** used in theoretical calculations 23
18. **Supplementary Figure 4**. Bond lengths and dihedral angle of **3** used in theoretical calculations 23
19. **Supplementary Figure 5**. X-ray crystal structures of **2** and **3** used in theoretical calculations 23
20. **Supplementary Figure 6**. 2D energy profiles for coordinatively-integrated and non-coordinatively integrated **2** and **3** 24
21. **Supplementary Figure 7**. X-ray crystal structure of **2** 24
22. **Supplementary Figure 8**. $^1$H NMR spectrum of **2** 25
23. **Supplementary Figure 9**. $^{13}$C NMR spectrum of **2** 25
24. **Supplementary Figure 10**. $^1$H NMR spectrum of **6** 26
25. **Supplementary Figure 11**. $^{13}$C NMR spectrum of **6** 26
26. **Supplementary Figure 12**. TGA plot of parent UiO-67 27
27. **Supplementary Figure 13**. $^1$H NMR spectrum of digested UiO-67+**2** 27
28. **Supplementary Figure 14**. $^1$H NMR spectrum of digested UiO-67+**5** 28
29. **Supplementary Figure 15**. $^1$H NMR spectrum of digested UiO-67+**6** 28

30. **Supplementary Figure 16**. $^1$H NMR spectrum of digested UiO-67+**11** 29
31. **Supplementary Figure 17**. $^1$H NMR spectrum of digested UiO-67+**2**+**5** 29
32. **Supplementary Figure 18**. PXRD patterns of UiO-67 and UiO-67+**2** before and after UV irradiation and evacuation 30
33. **Supplementary Figure 19**. PXRD patterns of UiO-67 and UiO-67+**5** before and after UV irradiation and evacuation 30
34. **Supplementary Figure 20**. PXRD patterns of UiO-67 and UiO-67+**6** before and after UV irradiation and evacuation 31
35. **Supplementary Figure 21**. PXRD patterns of UiO-67 and UiO-67+**11** before and after UV irradiation and evacuation 31
36. **Supplementary Figure 22**. PXRD patterns of UiO-67 and UiO-67+**2**+**5** before and after UV irradiation and evacuation 32
37. **Supplementary Figure 23**. PXRD patterns of $Zn_2$(**3**)(DBTD) before and after UV irradiation and evacuation 32
38. **Supplementary Figure 24**. PXRD patterns of $Zn_2$(**12**)(DBTD) before and after UV irradiation 33
39. **Supplementary Figure 25**. Absorbance spectra and photoisomerization kinetics studies of **1** in DMF 33
40. **Supplementary Figure 26**. Absorbance spectra and photoisomerization kinetics studies of **1** in ethanol 34
41. **Supplementary Figure 27**. Absorbance spectra and photoisomerization kinetics studies of **1** in THF 34
42. **Supplementary Figure 28**. Absorbance spectra and photoisomerization kinetics studies of **1** in toluene 35
43. **Supplementary Figure 29**. Absorbance spectra and photoisomerization kinetics studies of **2** in THF 35
44. **Supplementary Figure 30**. Absorbance spectra and photoisomerization kinetics studies of **3** in DMF 36
45. **Supplementary Figure 31**. Absorbance spectra and photoisomerization kinetics studies of **3** in ethanol 36
46. **Supplementary Figure 32**. Absorbance spectra and photoisomerization kinetics studies of **3** in acetonitrile 37
47. **Supplementary Figure 33**. Absorbance spectra and photoisomerization kinetics studies of **3** in toluene 37
48. **Supplementary Figure 34**. Diffuse reflectance spectra and photoisomerization kinetics studies of as-synthesized $Zn_2$(**3**)(DBTD) 38
49. **Supplementary Figure 35**. Diffuse reflectance spectra and photoisomerization kinetics studies of evacuated $Zn_2$(**3**)(DBTD) 38
50. **Supplementary Figure 36**. Absorbance spectra and photoisomerization kinetics studies of **4** in DMF 39
51. **Supplementary Figure 37**. Absorbance spectra and photoisomerization kinetics studies of **4** in toluene 39
52. **Supplementary Figure 38**. Absorbance spectra and photoisomerization kinetics studies of **5** in DMF 40
53. **Supplementary Figure 39**. Absorbance spectra and photoisomerization kinetics studies of **5** in ethanol 40

54. **Supplementary Figure 40**. Diffuse reflectance spectra and photoisomerization kinetics studies of as-synthesized UiO-67+**5** 41
55. **Supplementary Figure 41**. Diffuse reflectance spectra and photoisomerization kinetics studies of evacuated UiO-67+**5** 41
56. **Supplementary Figure 42**. Absorbance spectra and photoisomerization kinetics studies of **6** in DMF 42
57. **Supplementary Figure 43**. Diffuse reflectance spectra and photoisomerization kinetics studies of as-synthesized UiO-67+**6** 42
58. **Supplementary Figure 44**. Diffuse reflectance spectra and photoisomerization kinetics studies of evacuated UiO-67+**6** 43
59. **Supplementary Figure 45**. Absorbance spectra and photoisomerization kinetics studies of **7** in methanol 43
60. **Supplementary Figure 46**. Absorbance spectra and photoisomerization kinetics studies of **8** in methanol 44
61. **Supplementary Figure 47**. Absorbance spectra and photoisomerization kinetics studies of **9** in toluene 44
62. **Supplementary Figure 48**. Diffuse reflectance spectra and photoisomerization kinetics studies of **10** in the solid state 45
63. **Supplementary Figure 49**. Diffuse reflectance spectra and photoisomerization kinetics studies of **11** in the solid state 45
64. **Supplementary Figure 50**. Absorbance spectra and photoisomerization kinetics studies of **11** in DMF 46
65. **Supplementary Figure 51**. Diffuse reflectance spectra and photoisomerization kinetics studies of as-synthesized UiO-67+**11** 46
66. **Supplementary Figure 52**. Diffuse reflectance spectra and photoisomerization kinetics studies of evacuated UiO-67+**11** 47
67. **Supplementary Figure 53**. Absorbance spectra and photoisomerization kinetics studies of **12** in DMF 47
68. **Supplementary Figure 54**. Diffuse reflectance spectra and photoisomerization kinetics studies of **12** in the solid state 48
69. **Supplementary Figure 55**. Absorbance spectra and photoisomerization kinetics studies of **12** in ethanol 48
70. **Supplementary Figure 56**. Absorbance spectra and photoisomerization kinetics studies of **12** in toluene 49
71. **Supplementary Figure 57**. Diffuse reflectance spectra and photoisomerization kinetics studies of as-synthesized $Zn_2$(**12**)(DBTD) 49
72. **Supplementary Figure 58**. Absorbance spectra and photoisomerization kinetics studies of **13** in acetonitrile 50
73. **Supplementary Figure 59**. Diffuse reflectance spectra of evacuated UiO-67+**2**+**5** 50
74. **Supplementary Figure 60**. Epifluorescence microscopic images of UiO-67+**2**+**5** before and after exposure to UV irradiation 51
75. **Supplementary Figure 61**. EDX spectrum of UiO-67+**2**+**5** after coordination of copper cations 51
76. **Supplementary Figure 62**. Elemental mapping of UiO-67+**2**+**5** after coordination of copper cations 51

77. **Supplementary Figure 63**. X-ray crystal structure of UiO-67 52
78. **Supplementary Figure 64**. X-ray crystal structures of a diarylethene derivative demonstrating minimal structural rearrangement upon photoisomerization 53
79. **Supplementary Figure 65**. Electrostatic charge distributions of non-integrated and coordinatively-integrated **2** in the ground and excited states 54
80. Supplementary References 54

**Supplementary Note 1.**

**Materials.** 1,3,3-trimethylindolino-6'-nitrobenzopyrolospiran (**3**, >98%, TCI), 5-chloro-3-[2-(5-chloro-2-methylthien-3-yl)cycopent-1-en-1-yl]-2-methylthiophene (**10**, 95.04%, AmBeed), 4,4'-biphenyldicarboxylic acid (97%, Oakwood Chemical), 3-methyl-2-butanone (98%, BeanTown Chemical), 4-hydrazineyl benzoic acid (98.77%, AmBeed, Inc.), methyl trifluoromethanesulfonate (97%, Matrix Scientific), zirconium chloride (99.5%, Alfa Aesar), hydrochloric acid (ACS, Fisher Chemical), 2-hydroxy-5-nitrobenzaldehyde (98%, Oakwood Chemical), glacial acetic acid (ACS grade, BDH), ethanol (200 proof, Decon Laboratories, Inc.), diethyl ether (>99%, Sigma-Aldrich), hexane (ACS grade, BDH), isopropyl alcohol (ACS grade, BDH), acetone (ACS grade, Sigma-Aldrich), dichloromethane (ACS grade, Macron), and pyridine (99%, BeanTown Chemical), *N,N*-dimethylformamide (ACS grade, Oakwood Chemical), tetrahydrofuran (>99.8%, Fisher Chemical), methanol (>99.8%, Honeywell), toluene (>99.8%, Fisher Chemical), acetonitrile (99%, Fisher Chemical), chloroform-*d* (Cambridge Isotopes), dimethyl sulfoxide-$d_6$ (Cambridge Isotopes), and sulfuric acid-$d_2$ (Cambridge Isotopes) were used as received.

The compounds 1',3',3'-trimethyl-6-nitro-4',7'-di(pyridin-4-yl)spiro[chromene-2,2'-indoline] (**3**),[1] (*E*)-1-(3-methoxybenzylidene)-2-phenylhydrazine (**4**),[2] (*E*)-4-(2-(4-carboxybenzylidene)hydrazineyl)benzoic acid (**5**),[3] (*E*)-2-((2-(pyridin-2-yl)hydrazineylidene)methyl)pyridine (**7**), [4](*E*)-2-((2-phenylhydrazineylidene)methyl)pyridine (**8**),[4] ethyl (*Z*)-2-(4-(diphenylamino)phenyl)-2-(2-(pyridin-4-yl)hydrazineylidene)acetate (**9**),[5]4,4'-(cyclopent-1-ene-1,2-diyl)bis(5-methylthiophene-2-carboxylic acid) (**11**),[6] 1,2-bis(2-methyl-5-(pyridin-4-yl)thiophen-3-yl)cyclopent-1-ene (**12**),[1] **13**,[7] $Zr_6O_4(OH)_4(BPDC)_6$ ($H_2BPDC$ = [1,1'-biphenyl]-4,4'-dicarboxylic acid: UiO-67; UiO = University of Oslo),[8] $Zn_2$(**3**)(DBTD),[1] and $Zn_2$(**12**)(DBTD) ($H_4DBTD$ = $H_4DBTD$ = 3′,6′-dibromo-4′,5′-bis(4-carboxyphenyl)-[1,1′,2′,1″-terphenyl]-4,4″-dicarboxylic acid)[1] were synthesized based on modified literature procedures.

## Supplementary Methods.

**Synthesis of 2,3,3-trimethyl-3*H*-indole-5-carboxylic acid (I, Supplementary Fig. 1).** The compound 2,3,3-trimethyl-3*H*-indole-5-carboxylic acid was prepared based on a modified literature procedure.[9] To a 250-mL round bottom flask, 3-methyl-2-butanone (2.20 mL, 20.5 mmol) and 4-hydrazineyl benzoic acid (2.00 g, 13.1 mmol), and acetic acid (25 mL) were added and heated at reflux for one day. After cooling to room temperature, the solvent was evaporated under reduced pressure. Then, 100 mL of acetone was added to the flask, and the reaction mixture was sonicated for 30 minutes. The filtrate was collected, and the solvent was removed under reduced pressure. Next, 100 mL of dichloromethane was added to the flask, and the resulting reaction mixture was sonicated for 30 minutes. The filtrate was collected, and the solvent was removed under reduced pressure, affording a red solid (1.82 g, 68%). The collected $^1H$ NMR spectrum matched the reported one[9] (DMSO-$d_6$, 300 MHz): $\delta$ = 12.78 (s, 1H), 7.99 (s, 1H), 7.93–7.89 (d, 1H), 7.52–7.49 (d, 1H), 2.25 (s, 3H), and 1.27 (s, 6H) ppm.

**Synthesis of [5-carboxy-1,2,3,3-tetramethyl-3*H*-indol-1-ium] [trifluoromethanesulfonate] (II, Supplementary Fig. 1).**

To a 250-mL round bottom flask, containing 100 mL of a mixture of diethyl ether and hexane (3:2 v/v), **I** (Supplementary Fig. 1, 1.00 g, 4.92 mmol), and methyl trifluoromethanesulfonate (1.37 mL, 12.5 mmol) were added. The resulting mixture was allowed to stir at room temperature for one day. Upon reaction completion, the precipitate was collected by vacuum filtration. The solid precipitate was transferred into a 250-mL round bottom flask, and then 100 mL of diethyl ether was added to the same flask. The resulting mixture was sonicated for 30 minutes to dissolve potential impurities. The precipitate was collected by vacuum filtration. The solid precipitate was transferred into a 250-mL round bottom flask, and 100 mL of isopropyl alcohol was added to the flask. The resulting mixture was sonicated for additional 30 minutes. The precipitate was collected by vacuum filtration, affording a solid (1.57 g, 87%). The collected $^{1}H$ NMR spectrum matched the reported spectrum[9] (DMSO-$d_6$, 400 MHz): $\delta$ = 8.38 (s, 1H), 8.21–8.18 (d, 1H), 8.03–8.00 (d, 1H), 3.98 (s, 3H), 2.79 (s, 3H), and 1.56 (s, 6H) ppm.

**Synthesis of 1',3',3'-trimethyl-6-nitrospiro[chromene-2,2'-indoline]-5'-carboxylic acid (2).**

To a 100-mL round bottom flask, **II** (Supplementary Fig. 1, 1.20 g, 3.27 mmol), 2-hydroxy-5-nitrobenzaldehyde (1.00 g, 5.98 mmol), pyridine (1.20 mL), and ethanol (15 mL) were added, and the reaction mixture was refluxed for one day under nitrogen. After cooling to room temperature, 50 mL of diethyl ether was added to the solution, and the resulting mixture was stirred for one hour. The precipitate was collected via filtration and washed with hexane, affording a solid (0.980 g, 82%). $^{1}H$ NMR (DMSO-$d_6$): $\delta$ = 12.35 (s, 1H), 8.24 (s, 1H), 8.03–8.00 (d, 1H), 7.83–7.80 (d, 1H), 7.68 (s, 1H), 7.27–7.24 (d, 1H), 6.92–6.90 (d, 1H), 6.70–6.68 (d,1H), 6.03–6.00 (d, 1H), 2.76 (s, 3H), 1.24 (s, 3H), and 1.13 (s, 3H) ppm. $^{13}C$ NMR (DMSO-$d_6$): $\delta$ = 167.37, 158.96, 151.20, 140.72, 135.88, 130.83, 128.50, 125.80, 122.94, 122.88, 121.60, 120.88, 118.78, 115.47, 106.27, 105.90, 51.56, 28.39, 25.48, and 19.53 ppm. Melting point: 285–287 °C. HR-ESI-MS: m/z found [M+H] for $C_{20}H_{19}N_2O_5^+$ 367.1290 (calculated 367.1294).

**Synthesis of 4-(2-(1-(4-carboxyphenyl)-2-ethoxy-2-oxoethylidene)hydrazineyl)benzoic acid (6).**

To a 25-mL round bottom flask, 4-(2-ethoxy-2-oxoacetyl)benzoic acid (50.0 mg, 0.230 mmol) and 4-hydrazinobenzoic acid (41.0 mg, 0.270 mmol) were added and dissolved in 5.00 mL of ethanol. The resulting mixture was then stirred at room temperature for eight hours. After eight hours, the reaction mixture was diluted with 10.0 mL of water. The resulting precipitate was collected by filtration, and the solid was purified by column chromatography (0–50% ethyl acetate to hexanes), affording **6** as a yellow solid (49.0 mg, 62%). $^{1}H$ NMR (DMSO-$d_6$) $\delta$ = 11.77 (s, 1H), 7.97 (d, J = 8.1 Hz, 2H), 7.91 (d, J = 8.4 Hz, 2H), 7.79 (d, J = 8.1 Hz, 2H), 7.44 (d, J = 8.4 Hz, 2H), 4.40 (q, J = 7.1 Hz, 2H), 1.32 (t, J = 7.1 Hz, 3H). $^{13}C$ NMR (DMSO-$d_6$) $\delta$ =167.53, 162.92, 147.45, 139.74, 131.65, 131.52, 130.59, 129.67, 127.95, 124.25, 114.07, 62.13, and 14.31. Melting point: 272–273 °C. HR-ESI-MS: m/z found [M+H] for $C_{18}H_{17}N_2O_6^+$ 357.1079 (calculated 357.1087).

**Preparation of UiO-67+2.**

To postsynthetically integrate **2** into UiO-67, parent UiO-67 was first synthesized based on a literature procedure.[8] The UiO-67 powder was collected by filtration, thoroughly washed with DMF (3 × 10 mL), and then dried in air for 10 minutes. After drying, UiO-67 (25.0 mg, 11.8 μmol) was placed in a 20-mL vial. Then, 1.00 mL of a 30.0 mM DMF solution of **2** was added. The vial containing the resulting mixture was placed in a preheated aluminum block at 75 °C for 24 hours. After cooling to room temperature, the resulting powder was collected by filtration and washed with DMF (3 × 10 mL). The PXRD patterns of parent UiO-67 and UiO-67+**2** are shown in Supplementary Fig. 18. PXRD studies confirmed that MOF crystallinity was preserved after postsynthetic linker installation. The amount of **2** integrated in UiO-67 was calculated using a combination of TGA of the MOF and $^{1}H$ NMR spectroscopic analysis of the digested sample, which corresponds to 0.01 of **2** integrated per metal node. The $^{1}H$ NMR spectrum of the digested MOF sample confirming integration of **2** is shown in Supplementary Fig. 13.

**Preparation of UiO-67+5.**

To postsynthetically integrate **5** in UiO-67, parent UiO-67 was first synthesized based on a literature procedure.[8] The UiO-67 powder was collected by filtration, thoroughly washed with DMF (3 × 10 mL), and then dried in air for 10 minutes. After drying, UiO-67 (25.0 mg, 11.8 μmol) was placed in a 20-mL vial. Then, 1.00 mL of a 30.0 mM DMF solution of **5** (1.00 mL) was added. The vial containing the resulting mixture was placed in a preheated aluminum block at 75 °C for 24 hours. After cooling to room temperature, the resulting powder was collected by filtration and washed with DMF (3 × 10 mL). The PXRD patterns of parent UiO-67 and UiO-67+**5** are shown in Supplementary Fig. 19. PXRD studies confirmed that MOF crystallinity was preserved after postsynthetic linker installation. The amount of **5** installed in UiO-67 was calculated using a combination of TGA of the MOF and $^{1}H$ NMR spectroscopic analysis of the digested sample, which corresponds to 0.4 of **5** integrated per metal node. The $^{1}H$ NMR spectrum of the digested MOF sample confirming integration of **5** is shown in Supplementary Fig. 14.

**Preparation of UiO-67+6.**

To postsynthetically integrate **6** in UiO-67, parent UiO-67 was first synthesized based on a literature procedure.[8] The UiO-67 powder was collected by filtration, thoroughly washed with DMF (3 × 10 mL), and then dried in air for 10 minutes. After drying, UiO-67 (25.0 mg, 11.8 μmol) was placed in a 20-mL vial. Then, 1.00 mL of a 30.0 mM DMF solution of **6** (1.00 mL) was added. The vial containing the resulting mixture was placed in a preheated aluminum block at 75 °C for 24 hours. After cooling to room temperature, the resulting powder was collected by filtration and washed with DMF (3 × 10 mL). The PXRD patterns of parent UiO-67 and UiO-67+**6** are shown in Supplementary Fig. 20. PXRD studies confirmed that MOF crystallinity was preserved after postsynthetic linker integration. The amount of **6** installed in UiO-67 was calculated using a combination of TGA of the MOF and $^{1}H$ NMR spectroscopic analysis of the digested sample, which corresponds to 0.4 of **6** integrated per metal node. The $^{1}H$ NMR spectrum of the digested MOF sample confirming integration of **6** is shown in Supplementary Fig. 15.

**Preparation of UiO-67+11.**

To postsynthetically integrate **11** in UiO-67, parent UiO-67 was first synthesized based on a literature procedure.[8] The UiO-67 powder was collected by filtration, thoroughly washed with DMF (3 × 10 mL), and then dried in air for 10 minutes. After drying, UiO-67 (25.0 mg, 11.8 μmol) was placed in a 20-mL vial. Then, 1.00 mL of a 30.0 mM DMF solution of **11** (1.00 mL) was added. The vial containing the resulting mixture was placed in a preheated aluminum block at 75 °C for 24 hours. After cooling to room temperature, the resulting powder was collected by filtration and washed with DMF (3 × 10 mL). The PXRD patterns of parent UiO-67 and UiO-67+**11** are shown in Supplementary Fig. 21. PXRD studies confirmed that MOF crystallinity was preserved after postsynthetic linker integration. The amount of **11** installed in UiO-67 was calculated using a combination of TGA of the MOF and $^1$H NMR spectroscopic analysis of the digested sample, which corresponds to 1.0 of **11** integrated per metal node. The $^1$H NMR spectrum of the digested MOF sample confirming integration of **11** is shown in Supplementary Fig. 16.

**Preparation of UiO-67+2+5.**

To postsynthetically integrate both **2** and **5** in UiO-67, parent UiO-67 was first synthesized based on a literature procedure.[8] The UiO-67 powder was collected by filtration, thoroughly washed with DMF (3 × 10 mL), and then dried in air for 10 minutes. After drying, UiO-67 (25.0 mg, 11.8 μmol) was placed in a 20-mL vial. Then, 0.500 mL of a 30.0 mM DMF solution of **5** was added, and the mixture was heated at 75 °C for two hours. After two hours, 0.500 mL of a 30.0 mM DMF solution of **2** was added. The vial containing the resulting mixture was heated at 75 °C for 24 hours. After cooling to room temperature, the resulting powder was collected by filtration and washed with DMF (3 × 10 mL). The PXRD patterns of parent UiO-67 and UiO-67+**2**+**5** are shown in Supplementary Fig. 22. PXRD studies confirmed that MOF crystallinity was preserved after postsynthetic linker integration. The amount of **2** and **5** installed in UiO-67 was calculated using a combination of TGA of the MOF and $^1$H NMR spectroscopic analysis of the digested sample, which corresponds to 0.1 of **2** and 0.6 of **5** integrated per metal node. The $^1$H NMR spectrum of the digested MOF sample confirming integration of both **2** and **5** is shown in Supplementary Fig. 17.

**General digestion procedure.**

For analysis by $^1$H NMR spectroscopy, UiO-67 samples with integrated photochromic molecules (2.00 mg) were transferred to a 20-mL vial and treated with 50.0 μL of $D_2SO_4$. The resulting solution was then diluted with 500 μL of DMSO-$d_6$. The samples were then sonicated for 10 minutes to ensure complete dissolution before being transferred to NMR tubes for analysis. The $^1$H NMR spectra of digested samples are shown in Supplementary Fig. 13–17.

**UV-vis absorbance spectroscopy.**

UV-vis absorbance spectra of hydrazone-containing samples in solution were collected using a ThermoFisher Evolution 350 UV-vis spectrometer with quartz cuvettes. Samples were irradiated with a mounted high-powered LED (M365L2, Thorlabs, $\lambda_{ex}$ = 365 nm, distance = 2.5 cm, and LEDD1B power supply set at 700 mA).

**Diffuse reflectance spectroscopy.**
Diffuse reflectance spectra for spiropyran- and diarylethene-containing samples were collected using an Ocean Optics JAZ spectrometer. An Ocean Optics ISP-REF integrating sphere was connected to the spectrometer using a 450-μm SMA fiber optic cable. For studies conducted under ambient atmosphere, the sample was loaded inside a 4.0-mm quartz sample holder. For studies conducted under dynamic vacuum, the sample was loaded into a quartz flow cell and connected to a Schlenk line with tygon tubing. In all cases, a 400-nm longpass glass filter (Thorlabs, FGL400) was placed between the quartz sample holder and the integrating sphere to filter any UV light from the internal tungsten-halogen lamp. The quartz sample holder and longpass filter were attached to the top of the integrating sphere with electrical tape to prevent sample displacement. A mounted high-powered LED (M365L2, Thorlabs, $\lambda_{ex}$ = 365 nm, distance = 2.5 cm, and LEDD1B power supply set at 700 mA) was used for *in situ* irradiation of the samples. Diffuse reflectance spectra of hydrazone-containing samples were collected using a ThermoFisher Evolution 350 UV-vis spectrometer paired with Harrick Scientific Praying Mantis Diffuse Reflection accessory. For studies conducted under ambient atmosphere, the samples were prepared by filling the sample holder with $BaSO_4$ and placing 1–2 mg of the hydrazone-containing sample on top of the $BaSO_4$ layer. For studies conducted under vacuum, the samples were loaded into a high-temperature reaction chamber (Part HVC-VUV-5) and evacuated for 24 hours on a Schlenk line prior to collection of the spectra. A mounted high-powered LED (M365L2, Thorlabs, $\lambda_{ex}$ = 365 nm, distance = 2.5 cm, and LEDD1B power supply set at 700 mA) was used for *in situ* irradiation of the samples.

**Thermogravimetric analysis.**
TGA was used to determine the thermal stability of the synthesized UiO-67 and estimate the number of defects per metal node.[10] TGA was performed on an SDT Q600 thermogravimetric analyzer. Samples were loaded on an alumina boat as the sample holder at a heating rate of 5 °C/minute to 600 °C under 10 mL/minute air flow. A significant weight loss was observed during heating above 150 °C (Supplementary Fig. 12), which was used to calculate the number of defects per metal node based on a literature procedure.[10]

**Epifluorescence microscopy**.
Epifluorescence microscopic images of UiO-67+**2**+**5** were collected using an Olympus BX51 microscope. Samples were irradiated for 30 s with a mounted high-powered LED (M365L2, Thorlabs, $\lambda_{ex}$ = 365 nm, distance = 2.5 cm, and LEDD1B power supply set at 700 mA). Images of the samples before and after UV-irradiation (Supplementary Fig. 57) were collected with a color digital CMOS camera (Canon EOS REBEL T3/1100D).

**Scanning electron microscopy and energy dispersive X-ray spectroscopy (SEM-EDX).**
To evaluate the distribution of photochromic molecules integrated into UiO-67, a 5.00-mg sample of UiO-67+**2**+**5** was exposed to 2.00 mL of a solution of $Cu(NO_3)_2$ (0.60 mM in DMF) under UV light ($\lambda_{ex}$ = 365 nm) for three days to promote coordination of copper cations by merocyanine.[6] After three days, the sample was washed thoroughly with DMF

and ethanol. Following the washing procedure, the sample was dried on vacuum overnight. The sample was mounted on a carbon substrate and analyzed with a Tescan Vega 3 SBU variable pressure SEM equipped with a backscattered electron detector. EDX analysis was performed with an accelerating voltage of 20.0 kV, a beam intensity of 13, a working distance of 10 mm, and 1962× magnification. EDX analysis revealed an equal distribution of copper in the sample (Supplementary Fig. 60). The obtained value for copper cation integration is in line with the amount of installed spiropyran. Based on elemental mapping, the copper cations bound by merocyanine and coordinated to remaining defect sites were evenly distributed throughout the bulk material (Supplementary Fig. 61).

**Other physical measurements.**
NMR spectra were obtained on Bruker Avance III-HD 300, Bruker Avance III 400 MHz, Oxford 500 MHz, and Bruker Ascend 600 MHz NMR spectrometers. $^{13}C$ and $^{1}H$ NMR spectra were referenced to natural abundance $^{13}C$ peaks and residual $^{1}H$ peaks of deuterated solvents, respectively. Chemical shifts are quoted in ppm relative to tetramethylsilane (TMS), using the residual solvent peak as the reference standard. FTIR spectra were collected on a Perkin-Elmer Spectrum 100. PXRD patterns were recorded on a Rigaku Miniflex II diffractometer at a scan rate of 5°/minute with accelerating voltage and current of 40 kV and 15 mA, respectively.

**Supplementary Note 2.**
*Computational details*.
*Initial model design*. For this computational analysis, we employed Q-Chem 5.4 software and utilized time-dependent density functional theory (TD-DFT) with the B3LYP functional and 6-31G* basis set. The initial geometries of **2** and **3**[1] (main text Figure 2) were derived from single-crystal X-ray analysis. To obtain optimized geometries of the non-coordinatively immobilized photochromic linkers at their ground states ($S_0$), we conducted geometry optimization without imposing any constraints and the resulting optimized geometries were extracted.

*Excited state geometry optimization*. The energy barrier was determined as the difference between the top of the energy barrier and the local minimum in the reactant region of excited state ($S_1$) potential energy surface. To calculate local minimum of the excited state energy surface, we performed the excited state geometry optimization for the previously optimized geometry without imposing any constraints. Subsequently, we continued the excited state geometry optimization of the photochromic linkers by stretching and constraining $r_{C-O}$ to obtain 2D energy profiles. Throughout this process, we observed a change in the dihedral angle (as defined in Supplementary Fig. 3 and 4) at each $r_{C-O}$. In the case of coordinatively integrated photochromic linkers, which were studied to model the behavior of the photochromic molecules after integration in the rigid MOF matrix, we fixed the atoms corresponding to the groups which can coordinate to the MOF metal nodes. For instance, the XYZ coordinates corresponding to the carboxylic acid group of **2** and pyridine groups of **3** were kept constant for the coordinatively integrated photochromic linkers. To constrain the highlighted atoms as shown in Supplementary Fig. 5, we used the XYZ coordinates from the minimum energy geometry of each derivative

as shown below. The coordinates which were kept constant for the coordinatively integrated photochromic molecules **2** and **3** are given below in Supplementary Tables 1 and 2**.**

*Excitation energy calculation*. The excited state energies were computed at stretched bond distances for both non-coordinatively integrated and coordinatively integrated photochromic molecules **2** and **3**, as described previously. These calculations allowed us to construct 2D energy profiles, which are illustrated in Supplementary Figure 6. In Supplementary Table 3, we provide the observed geometric parameters and excitation energy for both coordinatively and non-coordinatively integrated photochromic molecule **3** as an example. This table offers a comprehensive overview of the respective geometric characteristics and associated excitation energies for each type of photochromic molecule.

As demonstrated in the 2D potential energy profile for **2** (Supplementary Figure 6, left), an energy jump occurred just before reaching the maximum energy point (specifically, from $r_{C–O}$ = 1.770–1.775 Å). At these two points, we observed significant differences in $r^2_{C–O}$ (defined in main text Figure 4) and the dihedral angle. To precisely locate the energy barrier, we introduced $\boldsymbol{r}^2_{C–O}$ as the second reaction coordinate. By incorporating $r^2_{C–O}$ as an additional constraint, we generated three-dimensional (3D) potential energy surfaces, which are shown in Figure 5 of the main text. In this particular case, we utilized two constraints, $r^1_{C–O}$ and $r^2_{C–O}$, to accurately characterize the system's potential energy landscape and the associated reaction coordinates.

The calculated results from Supplementary Tables 4 and 5 were fitted to a quadratic form using Maple software in order to determine the saddle point for both non-coordinatively integrated and coordinatively integrated **2**. The fitted function and the estimated parameters are given by Supplementary Equation 1 and Supplementary Table 6 below.

$$f(r^1_{C-O}, r^2_{C-O}) = \frac{1}{2}a_{11}(r^1_{C-O})^2 + a_{12}r^1_{C-O}r^2_{C-O} + \frac{1}{2}a_{22}(r^2_{C-O})^2 + b_1 r^1_{C-O} + b_2 r^2_{C-O} + c_0 \quad \text{(Eq. 1)}$$

After determining the saddle points for both the non-coordinatively integrated and coordinatively integrated photochromic molecules from the quadratic fit, we performed the excited state geometry optimizations at the estimated values of $r^1_{C–O}$ and $r^2_{C–O}$. By calculating the energy difference between the saddle point and the local minima, we obtained the excitation energies. The calculated energies at each saddle point are provided in Supplementary Table 7. The geometric parameters and excitation energies for non-coordinatively integrated and coordinatively integrated **2** are given in Supplementary Table 8.

*Electrostatic charge distribution calculation*. Computational studies of solvent effects on the energetics of electronically excited states are difficult when significant charge rearrangement occurs upon excitation, as common computational approaches often use unrelaxed ground state density to compute the solvent response.[11] The problem is further complicated by the possible reordering of the excited states in the solvent, and the need to do a partial geometry optimization in the excited state. The comparison of electrostatic

potentials in the ground and excited states provides a qualitative measure of the change of interactions of a molecule with a solvent upon photoexcitation. Thus, we visualized the electrostatic charge distributions for non-integrated and coordinatively-integrated **2** in the ground and excited states, which were computed using TD-DFT at the B3LYP/6-31G* level (Supplementary Fig. 65). The molecules were visualized with VMD 1.9.4[12] at the density surface using an isovalue of 0.035, with the coloring of the electrostatic potential ranging from −0.08–0.20, all in atomic units.

**Supplementary Note 3.**
*X-ray crystal structure determination for* $C_{20}H_{18}N_2O_5$ *(2)*.

X-ray intensity data from a colorless plate were collected at 100(2) K using a Bruker D8 QUEST diffractometer equipped with a PHOTON-II area detector and an Incoatec microfocus source (Mo $K_\alpha$ radiation, $\lambda$ = 0.71073 Å). The raw area detector data frames were reduced, scaled, and corrected for absorption effects using the Bruker APEX3, SAINT+ and SADABS programs.[13,14] The structure was solved with SHELXT.[15,16] Subsequent difference Fourier calculations and full-matrix least-squares refinement against $F^2$ were performed with SHELXL-2018[15,16] using OLEX2.[17]

The compound crystallizes in the triclinic system. The space group *P*-1 (No. 2) was confirmed by structure solution. The asymmetric unit consists of two crystallographically independent, enantiomeric molecules. These were numbered similarly except for atom label suffixes A or B. All non-hydrogen atoms were refined with anisotropic displacement parameters. Hydrogen atoms bonded to carbon were located in difference Fourier maps before being placed in geometrically idealized positions and included as riding atoms with $d$(C–H) = 0.95 Å and $U_{iso}$(H) = 1.2$U_{eq}$(C) for arene hydrogen atoms and $d$(C–H) = 0.98 Å and $U_{iso}$(H) = 1.5$U_{eq}$(C) for methyl hydrogens. The methyl hydrogens were allowed to rotate as a rigid group to the orientation of maximum observed electron density. The two carboxylic acid group hydrogen atoms were located and refined freely. The largest residual electron density peak in the final difference map is 0.25 e/Å$^3$, located 0.88 Å from C3A.

**Supplementary Table 1**. The coordinates of the carboxylic acid group which was fixed to model the behavior of **2** integrated in the rigid MOF matrix.

| atom | *x* | *y* | *z* |
|---|---|---|---|
| C | 2.0795062688 | 4.8729843312 | 8.9040273999 |
| O | 2.1503442688 | 3.8196233312 | 9.5286913999 |
| O | 1.8567002688 | 6.0851663312 | 9.5889153999 |
| H | 1.7840672688 | 5.9124213312 | 10.5284093999 |

**Supplementary Table 2**. The coordinates of the pyridine groups which were fixed to model the behavior of **3** integrated in the rigid MOF matrix.

| atom | *x* | *y* | *z* |
|---|---|---|---|
| N | 2.1851223229 | 10.0122329146 | 3.4430717497 |
| N | 3.2228923229 | 0.1413759146 | 9.0839017497 |
| C | 2.3376913229 | 7.4826499146 | 4.6756157497 |
| C | 3.4479083229 | 8.0559979146 | 4.0570657497 |
| H | 4.2828533229 | 7.6031379146 | 4.0494357497 |
| C | 3.3146603229 | 9.2943629146 | 3.4546027497 |
| H | 4.0765143229 | 9.6597949146 | 3.0200237497 |
| C | 1.1480763229 | 9.4633569146 | 4.0754297497 |
| H | 0.3347203229 | 9.9548789146 | 4.1001297497 |
| C | 1.1729933229 | 8.2310309146 | 4.6966847497 |
| H | 0.3979903229 | 7.8991879146 | 5.1348367497 |
| C | 2.7010683229 | 2.4474329146 | 7.5504117497 |
| C | 3.7740913229 | 1.6069339146 | 7.2629887497 |
| H | 4.3606123229 | 1.8055549146 | 6.5426097497 |
| C | 3.9743763229 | 0.4828459146 | 8.0365667497 |
| H | 4.6987123229 | −0.0880260854 | 7.8075257497 |
| C | 2.2204193229 | 0.9771279146 | 9.3770187497 |
| H | 1.6817233229 | 0.7768669146 | 10.1332577497 |
| C | 1.9181093229 | 2.1145859146 | 8.6498497497 |
| H | 1.1830173229 | 2.6623789146 | 8.8992337497 |

**Supplementary Table 3**. Geometric parameters and excitation energies for uncoordinated and coordinatively integrated **3**.

| parameter | non-coordinatively integrated 3 | coordinatively integrated 3 |
|---|---|---|
| $r^1_{C–O}$ at $E_{min}$, Å | 1.386 | 1.400 |
| dihedral angle at $E_{min}$, ° | −98.14 | −137.75 |
| $r^1_{C–O}$ at $E_{max}$, Å | 1.790 | 1.800 |
| dihedral angle at $E_{max}$, ° | −166.90 | −144.56 |
| excitation energy, kcal/mol | 24.901 | 24.199 |

**Supplementary Table 4**. Calculated energies as a function of various $r^1_{C-O}$ and $r^2_{C-O}$ for non-coordinatively integrated **2**.

| $r^1_{C-O}$, Å | $r^2_{C-O}$, Å | energy, kcal/mol |
|---|---|---|
| 1.75 | 1.25 | −789629.4406 |
| 1.76 | 1.25 | −789629.9028 |
| 1.77 | 1.25 | −789630.3869 |
| 1.775 | 1.25 | −789630.6358 |
| 1.78 | 1.25 | −789630.8888 |
| 1.79 | 1.25 | −789631.4055 |
| 1.8 | 1.25 | −789631.9345 |
| 1.81 | 1.25 | −789632.4741 |
| 1.82 | 1.25 | −789633.0222 |
| 1.75 | 1.3 | −789632.489 |
| 1.76 | 1.3 | −789631.9988 |
| 1.77 | 1.3 | −789631.5676 |
| 1.775 | 1.3 | −789631.7126 |
| 1.78 | 1.3 | −789631.8744 |
| 1.79 | 1.3 | −789632.2305 |
| 1.8 | 1.3 | −789632.6194 |
| 1.81 | 1.3 | −789633.0327 |
| 1.82 | 1.3 | −789633.4647 |
| 1.79 | 1.305 | −789632.18 |
| 1.8 | 1.305 | −789632.5505 |
| 1.81 | 1.305 | −789632.9485 |
| 1.79 | 1.31 | −789632.1112 |
| 1.8 | 1.31 | −789632.462 |
| 1.81 | 1.31 | −789632.8452 |
| 1.78 | 1.315 | −789631.7569 |
| 1.785 | 1.315 | −789631.8813 |
| 1.79 | 1.315 | −789632.026 |
| 1.795 | 1.315 | −789632.1853 |
| 1.8 | 1.315 | −789632.3547 |
| 1.81 | 1.315 | −789632.7204 |
| 1.79 | 1.32 | −789631.9274 |
| 1.8 | 1.32 | −789632.2324 |
| 1.81 | 1.32 | −789632.5773 |
| 1.75 | 1.325 | −789634.9603 |
| 1.76 | 1.325 | −789634.1429 |
| 1.77 | 1.325 | −789632.7486 |
| 1.775 | 1.325 | −789632.4877 |
| 1.78 | 1.325 | −789632.2332 |
| 1.79 | 1.325 | −789631.8188 |
| 1.8 | 1.325 | −789632.0923 |

| | | |
|---|---|---|
| 1.81 | 1.325 | −789632.4189 |
| 1.82 | 1.325 | −789632.7771 |
| 1.75 | 1.35 | −789636.2786 |
| 1.76 | 1.35 | −789635.4446 |
| 1.77 | 1.35 | −789634.6067 |
| 1.775 | 1.35 | −789634.1895 |
| 1.78 | 1.35 | −789633.7725 |
| 1.79 | 1.35 | −789632.2862 |
| 1.8 | 1.35 | −789631.7524 |
| 1.81 | 1.35 | −789631.4143 |
| 1.82 | 1.35 | −789631.6607 |
| 1.75 | 1.375 | −789637.0209 |
| 1.76 | 1.375 | −789636.1685 |
| 1.77 | 1.375 | −789635.3204 |
| 1.775 | 1.375 | −789634.8944 |
| 1.78 | 1.375 | −789634.4695 |
| 1.79 | 1.375 | −789633.6187 |
| 1.8 | 1.375 | −789632.7695 |
| 1.81 | 1.375 | −789631.2086 |
| 1.82 | 1.375 | −789630.6636 |
| 1.75 | 1.4 | −789637.2645 |
| 1.76 | 1.4 | −789636.4007 |
| 1.77 | 1.4 | −789635.5373 |
| 1.775 | 1.4 | −789635.1045 |
| 1.78 | 1.4 | −789634.6711 |
| 1.79 | 1.4 | −789633.8096 |
| 1.8 | 1.4 | −789632.9497 |
| 1.81 | 1.4 | −789632.0881 |
| 1.82 | 1.4 | −789631.2357 |

**Supplementary Table 5**. Calculated energies as a function of various $r^1_{C–O}$ and $r^2_{C–O}$ for coordinatively-integrated **2**.

| $r^1_{C–O}$, Å | $r^2_{C–O}$, Å | energy, kcal/mol |
|---|---|---|
| 1.84 | 1.25 | −789631.6324 |
| 1.77 | 1.3 | −789628.9084 |
| 1.775 | 1.3 | −789629.0724 |
| 1.81 | 1.3 | −789630.4596 |
| 1.82 | 1.3 | −789630.9047 |
| 1.84 | 1.3 | −789631.859 |
| 1.77 | 1.31 | −789628.8969 |
| 1.775 | 1.31 | −789629.0004 |
| 1.81 | 1.31 | −789630.2669 |
| 1.82 | 1.31 | −789630.6853 |
| 1.84 | 1.31 | −789631.5866 |
| 1.77 | 1.316 | −789629.4201 |
| 1.775 | 1.316 | −789628.9584 |
| 1.78 | 1.316 | −789629.0846 |
| 1.785 | 1.316 | −789629.2294 |
| 1.79 | 1.316 | −789629.386 |
| 1.81 | 1.316 | −789630.1115 |
| 1.82 | 1.316 | −789630.5132 |
| 1.84 | 1.316 | −789631.379 |
| 1.77 | 1.317 | −789629.4657 |
| 1.775 | 1.317 | −789628.9504 |
| 1.78 | 1.317 | −789629.0345 |
| 1.785 | 1.317 | −789629.211 |
| 1.79 | 1.317 | −789629.3651 |
| 1.81 | 1.317 | −789630.053 |
| 1.82 | 1.317 | −789630.4817 |
| 1.84 | 1.317 | −789631.3425 |
| 1.77 | 1.318 | −789629.5104 |
| 1.775 | 1.318 | −789628.9378 |
| 1.78 | 1.318 | −789629.0466 |
| 1.785 | 1.318 | −789629.1921 |
| 1.79 | 1.318 | −789629.3436 |
| 1.81 | 1.318 | −789630.0236 |
| 1.82 | 1.318 | −789630.4495 |
| 1.84 | 1.318 | −789631.3052 |
| 1.77 | 1.319 | −789629.5554 |
| 1.775 | 1.319 | −789628.9455 |
| 1.78 | 1.319 | −789629.0334 |
| 1.785 | 1.319 | −789629.1728 |
| 1.79 | 1.319 | −789629.3215 |

| | | |
|---|---|---|
| 1.81 | 1.319 | −789629.9935 |
| 1.82 | 1.319 | −789630.4164 |
| 1.84 | 1.319 | −789631.2671 |
| 1.77 | 1.32 | −789629.558 |
| 1.775 | 1.32 | −789629.35 |
| 1.78 | 1.32 | −789629.0097 |
| 1.785 | 1.32 | −789629.1531 |
| 1.79 | 1.32 | −789629.2988 |
| 1.81 | 1.32 | −789629.9627 |
| 1.82 | 1.32 | −789630.3827 |
| 1.84 | 1.32 | −789631.2282 |
| 1.77 | 1.33 | −789629.9836 |
| 1.775 | 1.33 | −789631.1945 |
| 1.78 | 1.33 | −789629.45 |
| 1.785 | 1.33 | −789628.9286 |
| 1.82 | 1.33 | −789630.0055 |
| 1.84 | 1.33 | −789630.7899 |
| 1.77 | 1.34 | −789630.2698 |
| 1.775 | 1.34 | −789629.9755 |
| 1.78 | 1.34 | −789629.6897 |
| 1.81 | 1.34 | −789629.2084 |
| 1.82 | 1.34 | −789629.5604 |
| 1.84 | 1.34 | −789630.2895 |
| 1.77 | 1.35 | −789631.7288 |
| 1.775 | 1.35 | −789631.325 |
| 1.78 | 1.35 | −789629.9352 |
| 1.81 | 1.35 | −789628.7773 |
| 1.82 | 1.35 | −789629.0573 |
| 1.84 | 1.35 | −789629.725 |
| 1.81 | 1.36 | −789628.2726 |
| 1.82 | 1.36 | −789628.5064 |
| 1.84 | 1.36 | −789629.1036 |
| 1.8 | 1.37 | −789629.7815 |
| 1.81 | 1.37 | −789628.3254 |
| 1.82 | 1.37 | −789627.9059 |
| 1.84 | 1.37 | −789628.431 |

**Supplementary Table 6**. Estimated parameters for the non-coordinatively integrated and coordinatively integrated **2** based on Equation 1.

| parameter | non-coordinatively integrated 2 | coordinatively integrated 2 |
|---|---|---|
| $a_{11}$ | −6.0703 | −10.4735 |
| $a_{12}$ | 5.5261 | 25.1074 |
| $a_{22}$ | −2.4925 | −15.3128 |
| $b_1$ | 3.5634 | −14.4485 |
| $b_2$ | −6.5899 | −24.5205 |
| $c_0$ | −1257.2180 | −1229.3473 |
| $R^2$ | 0.9815 | 0.8578 |

**Supplementary Table 7**. Calculated energies at the saddle point of non-coordinatively integrated and coordinatively integrated **2**.

| parameter | non-coordinatively integrated 2 | coordinatively integrated 2 |
|---|---|---|
| energy at the saddle point, kcal/mol | −789631.8859 | −789629.1202 |
| $r^1_{C–O}$, Å | 1.787 | 1.781 |
| $r^2_{C–O}$, Å | 1.318 | 1.318 |

**Supplementary Table 8**. Geometric parameters and calculated excitation energies for non-coordinatively integrated and coordinatively integrated **2**.

| parameter | non-coordinatively integrated 2 | coordinatively integrated 2 |
|---|---|---|
| $r^1_{C–O}$ at $E_{min}$, Å | 1.399 | 1.392 |
| dihedral angle at $E_{min}$, ° | −143.13 | −124.37 |
| $r^2_{C–O}$ at $E_{min}$, Å | 1.420 | 1.419 |
| $r^1_{C–O}$ at $E_{max}$, Å | 1.787 | 1.781 |
| dihedral angle at $E_{max}$,° | −167.41 | −165.73 |
| $r^2_{C–O}$ at $E_{max}$, Å | 1.318 | 1.318 |
| excitation energy, kcal/mol | 26.655 | 26.571 |

**Supplementary Table 9**. X-ray structure refinement details for **2**.[a]

| compound | **2** |
|---|---|
| formula | $C_{20}H_{18}N_2O_5$ |
| FW, g mol$^{-1}$ | 366.36 |
| *T*, K | 100(2) |
| crystal system | triclinic |
| space group | *P-1* |
| Z | 4 |
| *a*, Å | 7.9047(6) |
| *b*, Å | 11.1546(9) |
| *c,* Å | 19.1569(15) |
| $\alpha$, ° | 94.061(2) |
| $\beta$, ° | 90.655(2) |
| $\gamma$, ° | 90.544(2) |
| *V*, Å$^3$ | 1684.7(2) |
| $d_{calc}$, g cm$^{-3}$ | 1.444 |
| μ, mm$^{-1}$ | 0.105 |
| F(000) | 768.0 |
| crystal size, mm$^3$ | 0.12 × 0.10 × 0.04 |
| 2 theta range | 3.66 to 48.58 |
| index ranges | $-9 \le h \le 9$<br>$-12 \le k \le 12$<br>$-22 \le l \le 22$ |
| reflections collected | 26701 |
| data/restraints/parameters | 5439/0/502 |
| GOF on $F^2$ | 1.070 |
| largest peak/hole, e/Å$^3$ | 0.25/–0.28 |
| $R_1$ ($wR_2$), %, [$I \ge$ 2sigma($I$)][c] | 6.95 (14.18) |

[a]Mo-K*α* ($\lambda$ = 0.71073 Å) radiation
[b]Formula is based on single crystal X-ray data and does not include disordered solvent molecules
[c]$R_1 = \Sigma||F_o| - |F_c||/ \Sigma|F_o|$, $wR_2 = \{\Sigma [w(F_o^2 - F_c^2)^2]/ \Sigma [w(F_o^2)^2]\}^{1/2}$

**Supplementary Table 10**. Comparison of literature photoisomerization rate constants and those obtained in this work.

| sample | conditions | $\lambda_{ex}$, nm | $k$, $s^{-1}$ | reference |
|---|---|---|---|---|
| **3** | 3 mM in DMF | 365 | $1.2 \times 10^{-1}$ | [1] |
| **3** | 3 mM in DMF | 365 | $0.8 \times 10^{-1}$ | this work |
| $Zn_2$(**3**)(DBTD) | solid state | 365 | $1.6 \times 10^{-1}$ | [1] |
| $Zn_2$(**3**)(DBTD) | solid state | 365 | $5.4 \times 10^{-2}$ | this work |
| **7** | 13 mM in $CD_3OD$ | 320–400 | $2.2 \times 10^{-3}$ | [4] |
| **7** | 10 µM in MeOH | 365 | $6.0 \times 10^{-4}$ | this work |
| **8** | 13 mM in $CD_3OD$ | 320–400 | $3.3 \times 10^{-4}$ | [4] |
| **8** | 10 µM in MeOH | 365 | $6.0 \times 10^{-4}$ | this work |
| **9** | 10 µM in toluene | 375 | $9.6 \times 10^{-2}$ | [5] |
| **9** | 10 µM in toluene | 365 | $3.0 \times 10^{-2}$ | this work |
| **11** | 3 mM in MeOH | 365 | $2.6 \times 10^{-2}$ | [6] |
| **11** | 3 mM in DMF | 365 | $1.9 \times 10^{-1}$ | this work |
| **11** | solid state | 365 | $4.8 \times 10^{-2}$ | [6] |
| **11** | solid state | 365 | $3.4 \times 10^{-2}$ | this work |
| **12** | 3 mM in DMF | 365 | $1.0 \times 10^{-2}$ | [1] |
| **12** | 3 mM in DMF | 365 | $3.7 \times 10^{-2}$ | this work |
| **12** | solid state | 365 | $2.0 \times 10^{-1}$ | [1] |
| **12** | solid state | 365 | $3.6 \times 10^{-1}$ | this work |
| $Zn_2$(**12**)(DBTD) | solid state | 365 | $2.8 \times 10^{-3}$ | [1] |
| $Zn_2$(**12**)(DBTD) | solid state | 365 | $3.3 \times 10^{-3}$ | this work |
| **13** | 50 µM in $CH_3CN$ | 365 | $7.0 \times 10^{-3}$ | [7] |
| **13** | 10 µM in $CH_3CN$ | 365 | $1.0 \times 10^{-2}$ | this work |

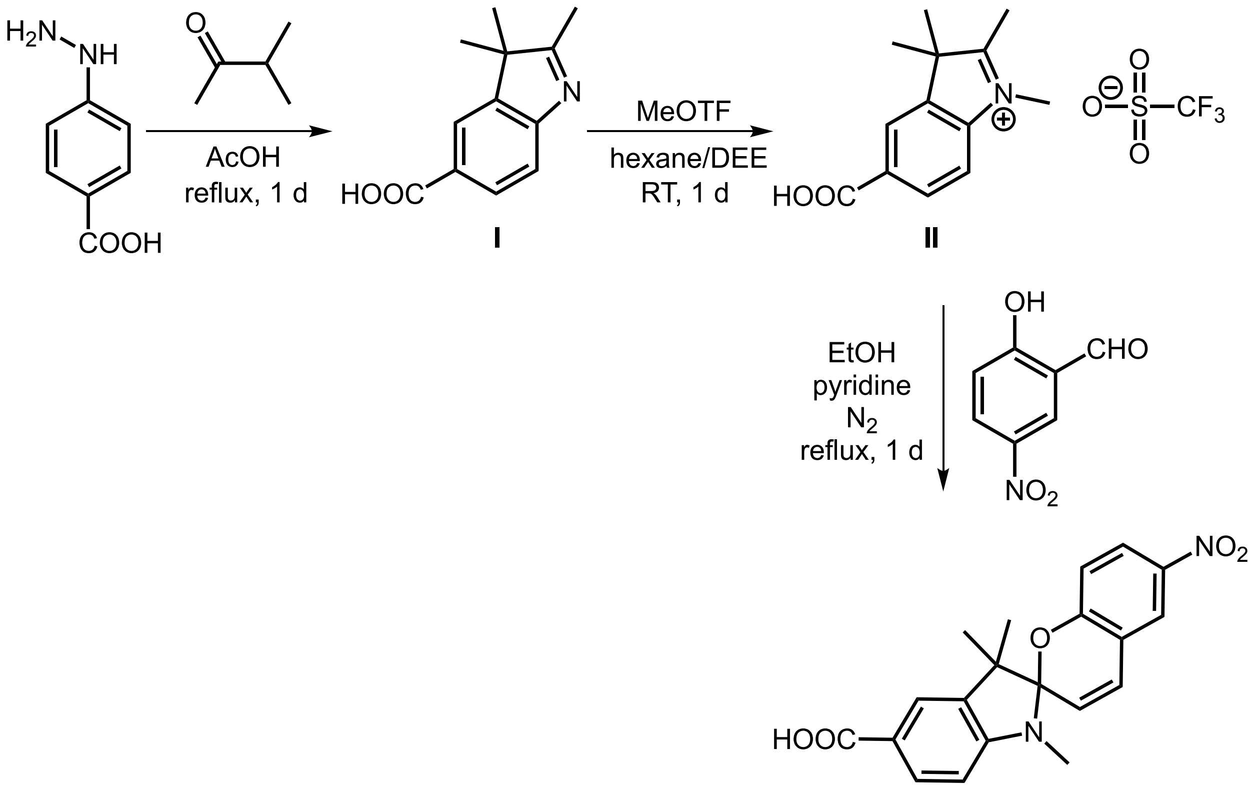


**2**

**Supplementary Figure 1**. Synthetic route for preparation of 1',3',3'-trimethyl-6-nitrospiro[chromene-2,2'-indoline]-5'-carboxylic acid (**2**).

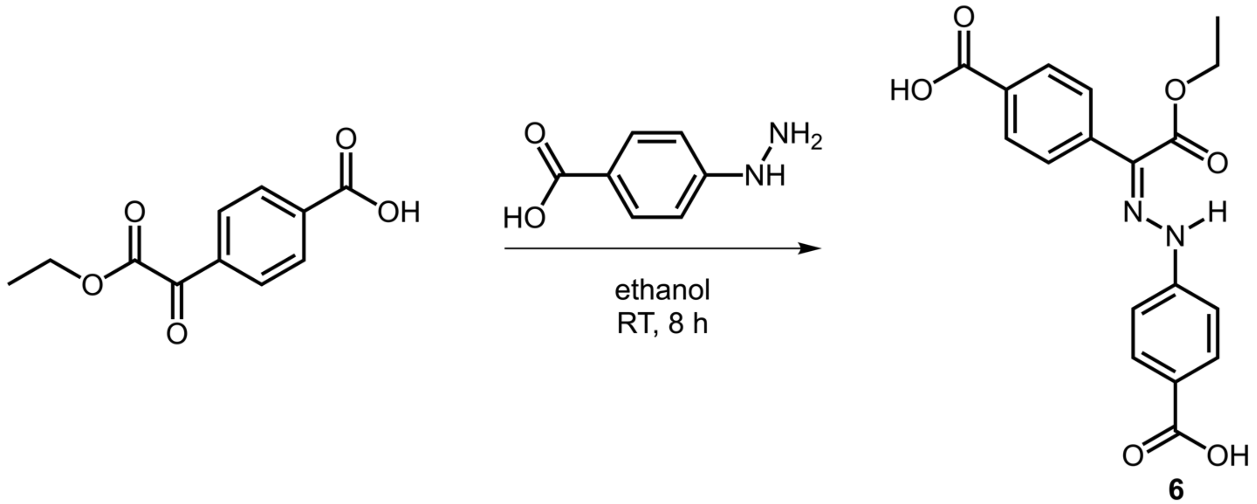


**6**

**Supplementary Figure 2**. Synthetic route for preparation of 4-(2-(1-(4-carboxyphenyl)-2-ethoxy-2-oxoethylidene)hydrazineyl)benzoic acid (**6**).

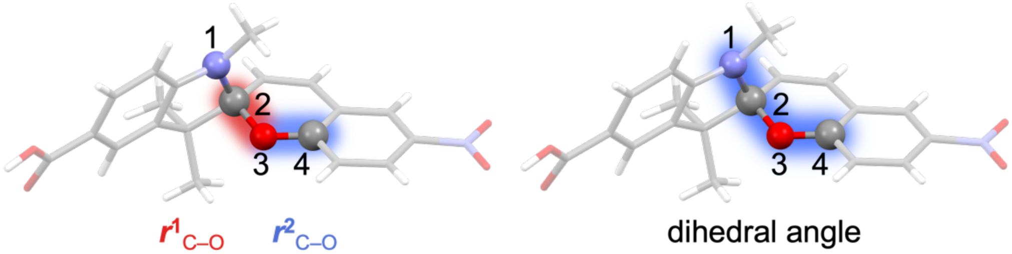


**Supplementary Figure 3**. X-ray crystal structure of **2** with the bond lengths (*left*) and dihedral angle (*right*) used as parameters in theoretical calculations highlighted. The bond length between atoms 2 and 3 is defined as $r^1_{C–O}$, while the bond length between atoms 3 and 4 is defined as $r^2_{C–O}$. The dihedral angle is defined by atoms 1–4.

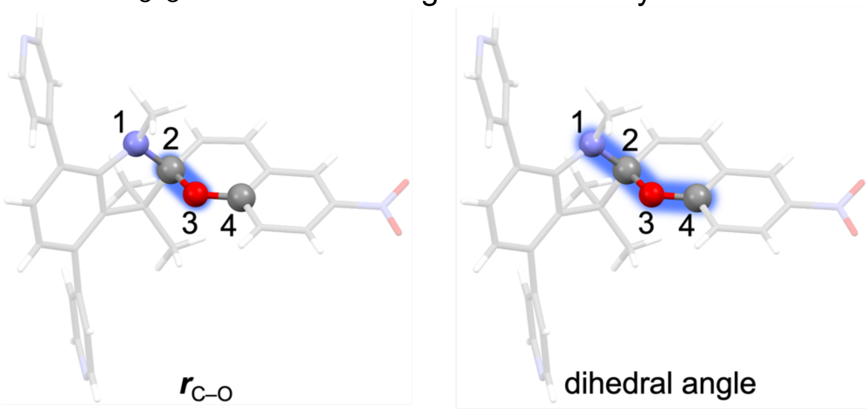


**Supplementary Figure 4**. X-ray crystal structure of **3** with the bond length (*left*) and dihedral angle (*right*) used as parameters in theoretical calculations highlighted. The bond length between atoms 2 and 3 is defined as $r_{C–O}$, and the dihedral angle is defined by atoms 1–4.

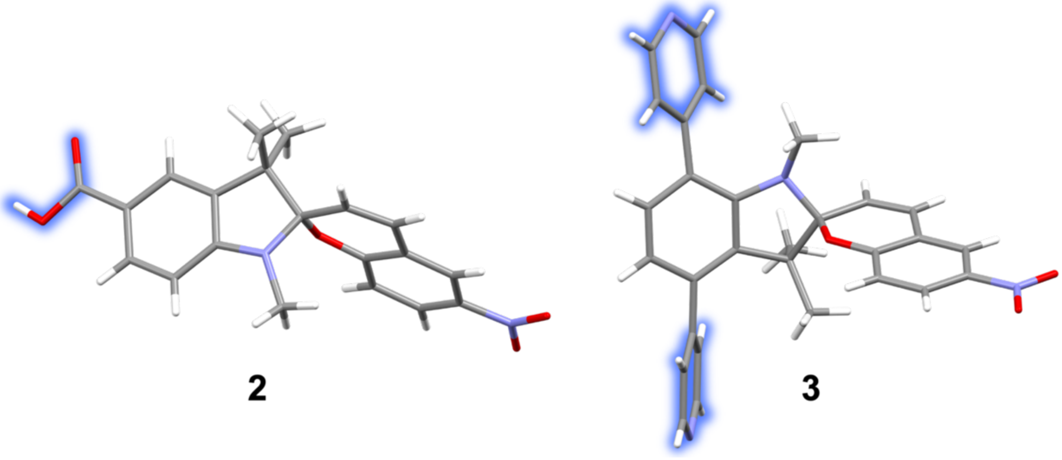


**Supplementary Figure 5**. X-ray crystal structures of **2** and **3** used as a starting point for excited state calculations. The atoms which were constrained for calculations are highlighted in blue.

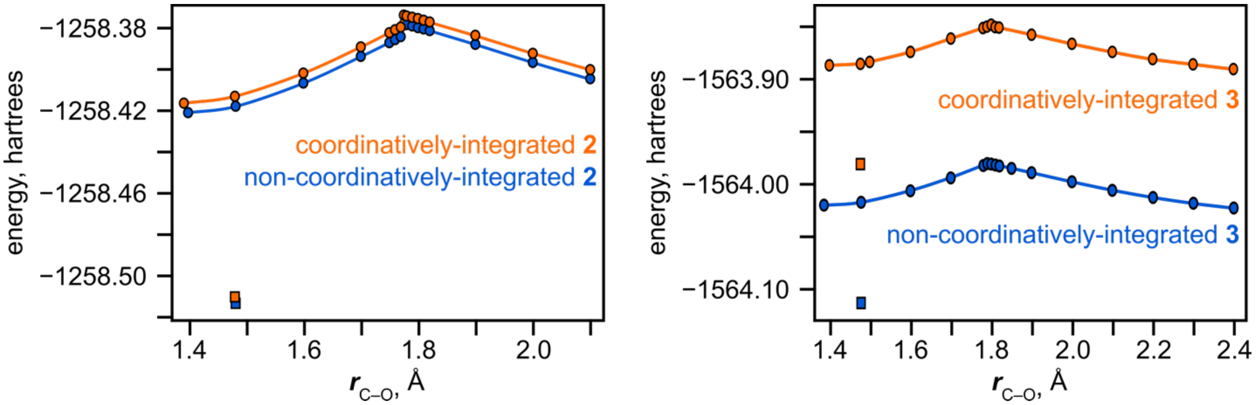


**Supplementary Figure 6**. (*left*) 2D energy profiles as a function of $r_{C–O}$ for non-coordinatively-integrated (blue) and coordinatively-integrated (orange) **2**. Both the excited state energies (circles) and optimized ground state energy (squares) are given. (*right*) 2D energy profiles as a function of $r_{C–O}$ for non-coordinatively-integrated (blue) and coordinatively-integrated (orange) **3**. Both the excited state energies (circles) and optimized ground state energy (squares) are given.

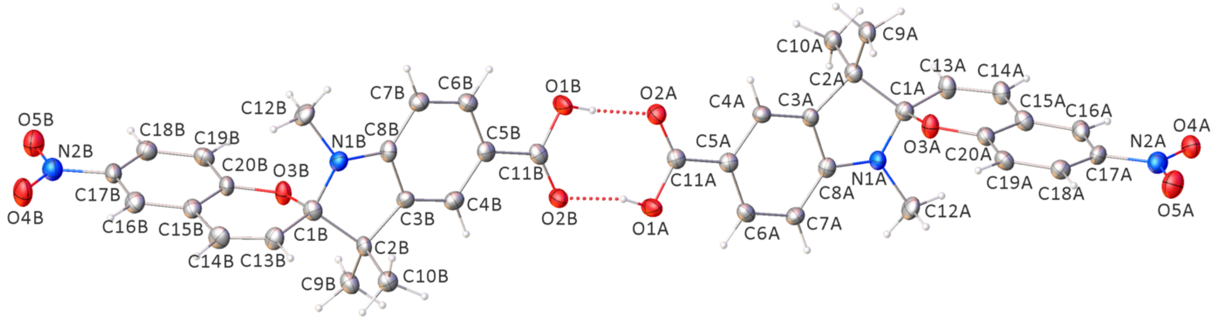


**Supplementary Figure 7**. X-ray crystal structure of **2**. Displacement ellipsoids are drawn at the 50% probability level.

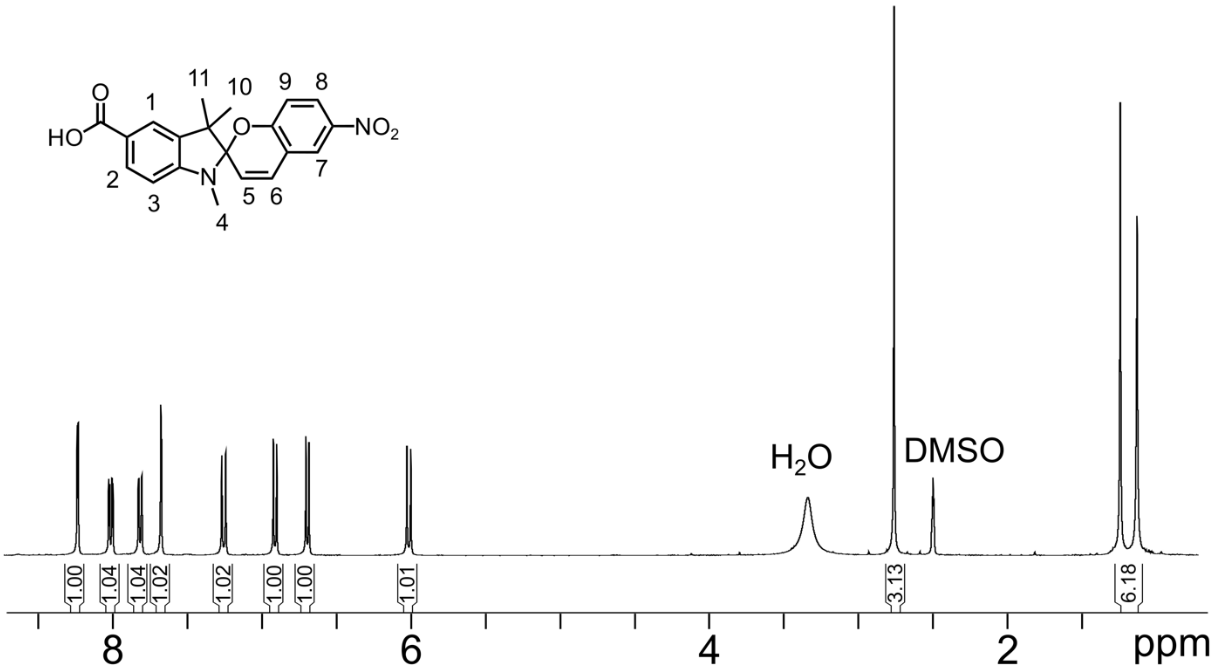


**Supplementary Figure 8**. $^{1}H$ NMR spectrum of **2** in DMSO-$d_6$ at 298 K.

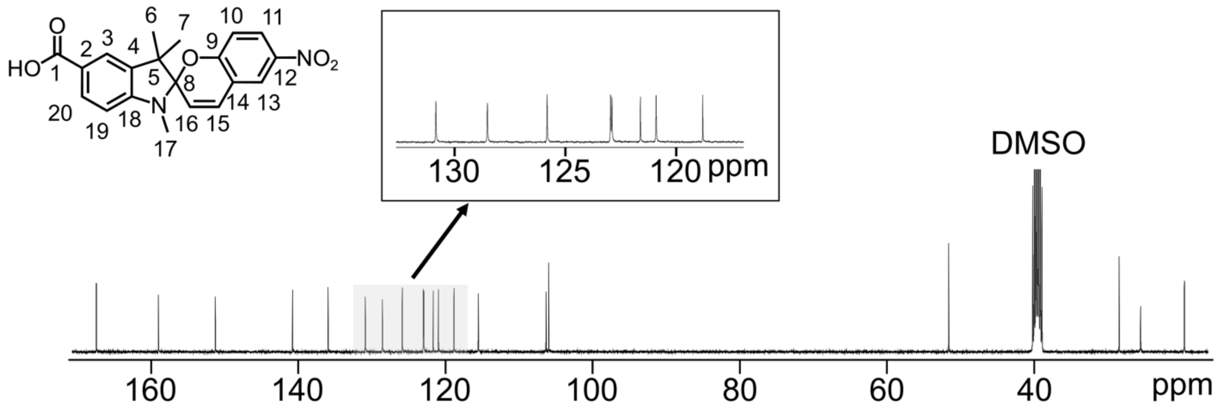


**Supplementary Figure 9**. $^{13}C$ NMR spectrum of **2** taken DMSO-$d_6$ at 298 K.

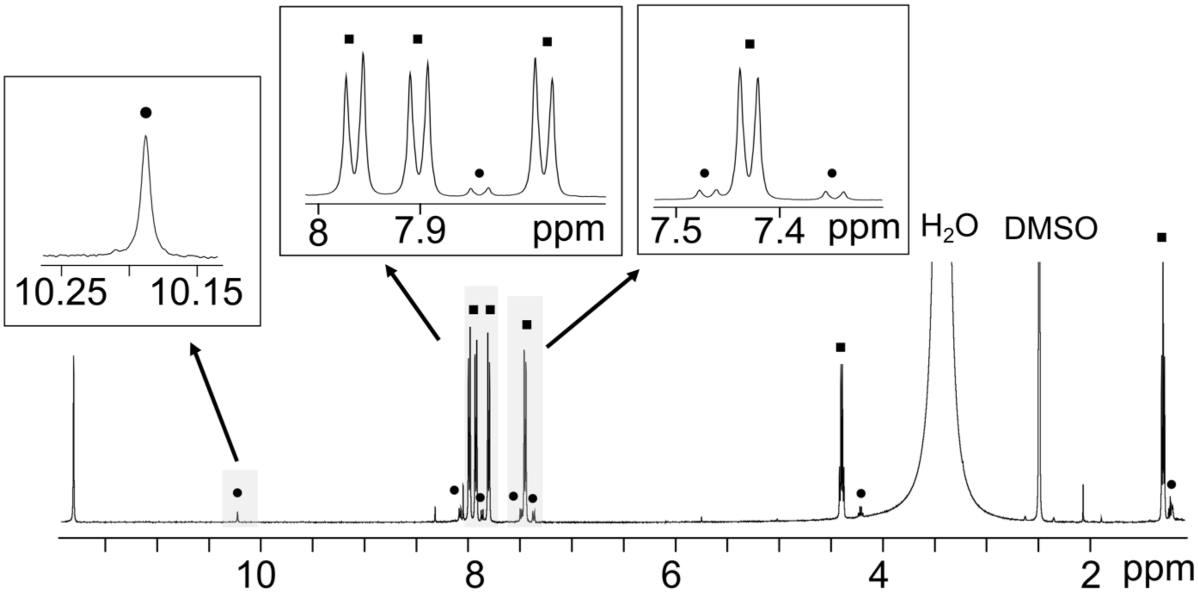


**Supplementary Figure 10**. $^{1}$H NMR spectrum of **6** in DMSO-$d_6$ at 298 K. Resonances corresponding to the *Z*-isomer (squares) and *E*-isomer (circles) are marked.

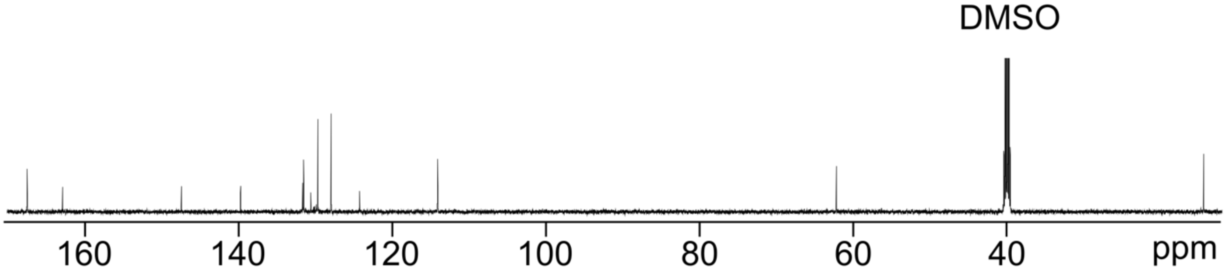


**Supplementary Figure 11**. $^{13}$C NMR spectrum of **6** taken DMSO-$d_6$ at 298 K.

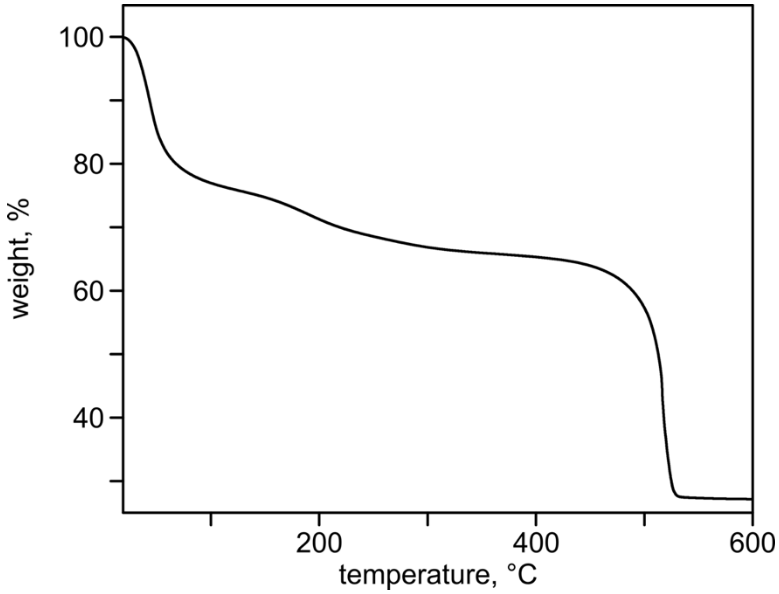


**Supplementary Figure 12**. TGA plot of the parent UiO-67 framework used for the photochromic MOF synthesis. The number of defects per metal node, which was calculated using a previously reported procedure,[10] was estimated to be 2.6.

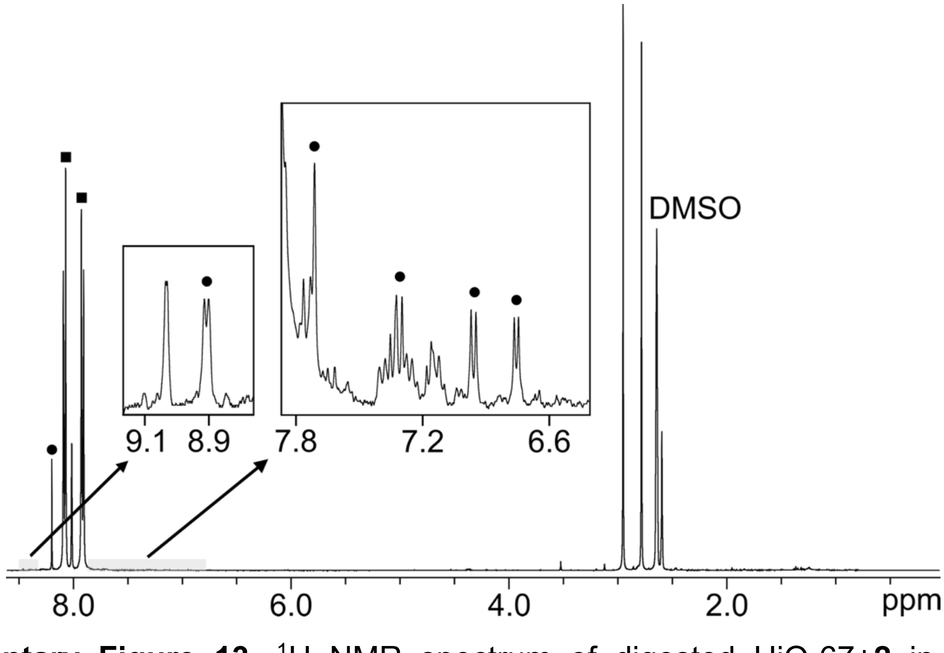


**Supplementary Figure 13**. $^1H$ NMR spectrum of digested UiO-67+**2** in DMSO-$d_6$. Resonances corresponding to **2** (circles) and the linker of UiO-67 (squares) are labeled.

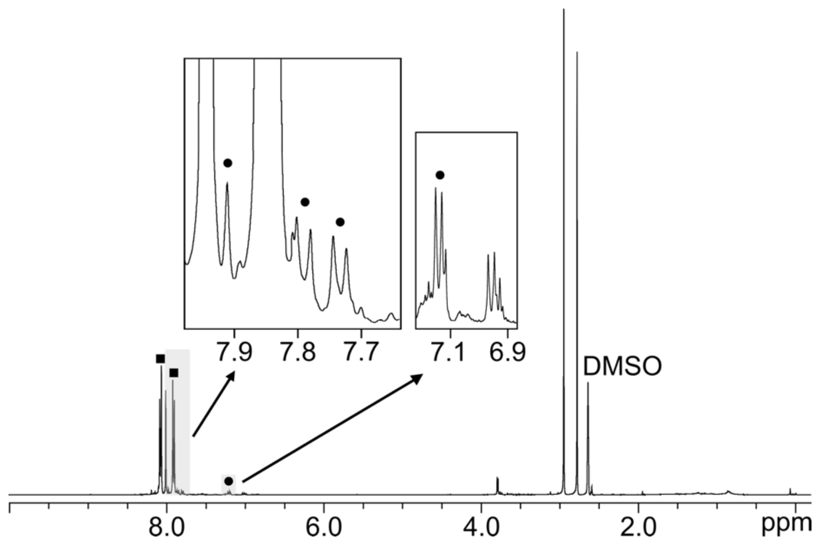


**Supplementary Figure 14**. $^1$H NMR spectrum of digested UiO-67+**5** in DMSO-$d_6$. Resonances corresponding to **5** (circles) and the linker of UiO-67 (squares) are labeled.

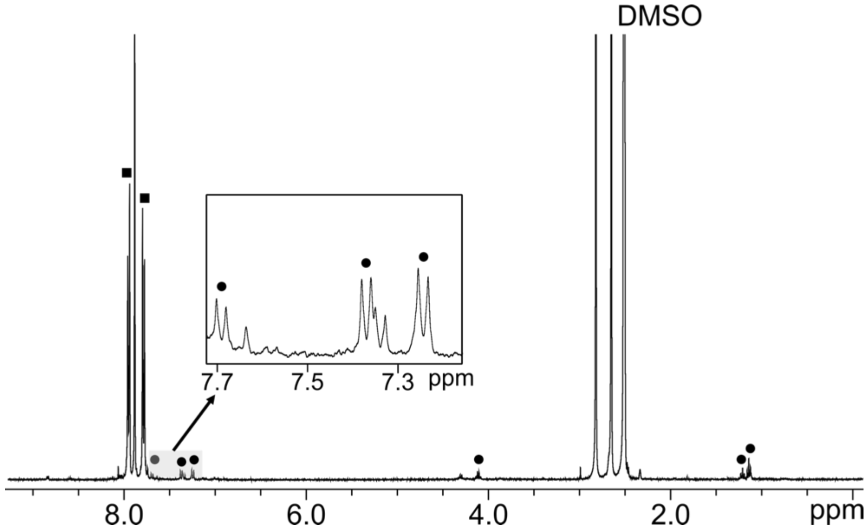


**Supplementary Figure 15**. $^1$H NMR spectrum of digested UiO-67+**6** in DMSO-$d_6$. Resonances corresponding to **6** (circles) and the linker of UiO-67 (squares) are labeled.

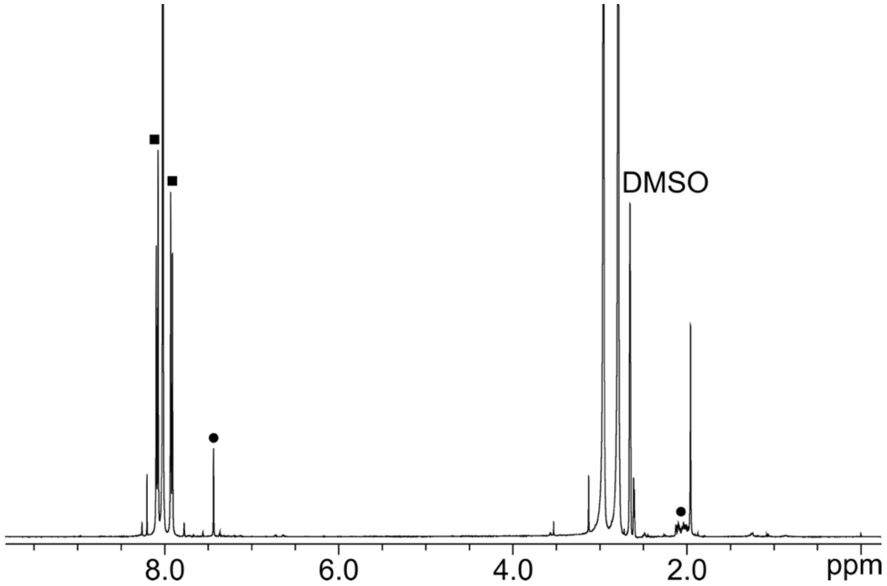


**Supplementary Figure 16**. $^{1}$H NMR spectrum of digested UiO-67+**11** in DMSO-$d_6$. Resonances corresponding to **11** (circles) and the linker of UiO-67 (squares) are labeled.

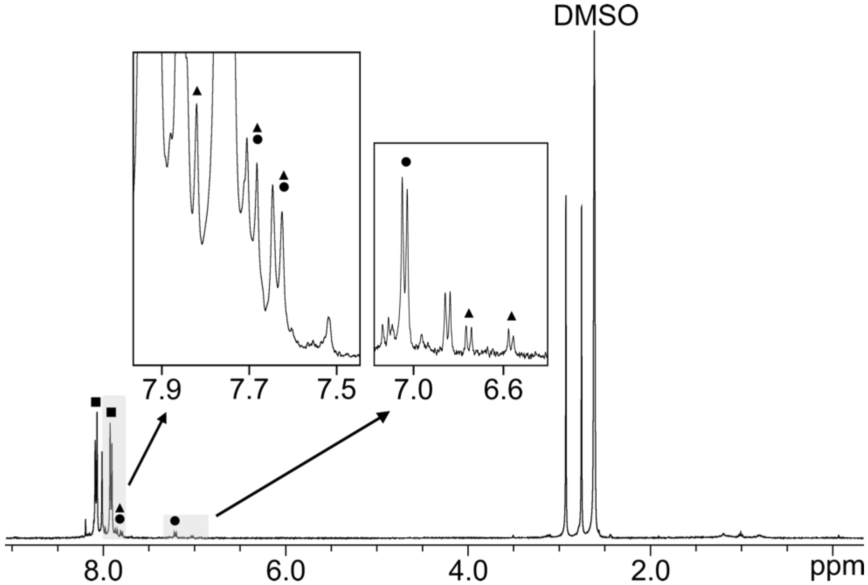


**Supplementary Figure 17**. $^{1}$H NMR spectrum of digested UiO-67+**2**+**5** in DMSO-$d_6$. Resonances corresponding to **2** (triangles), **5** (circles), and the linker of UiO-67 (squares) are labeled.

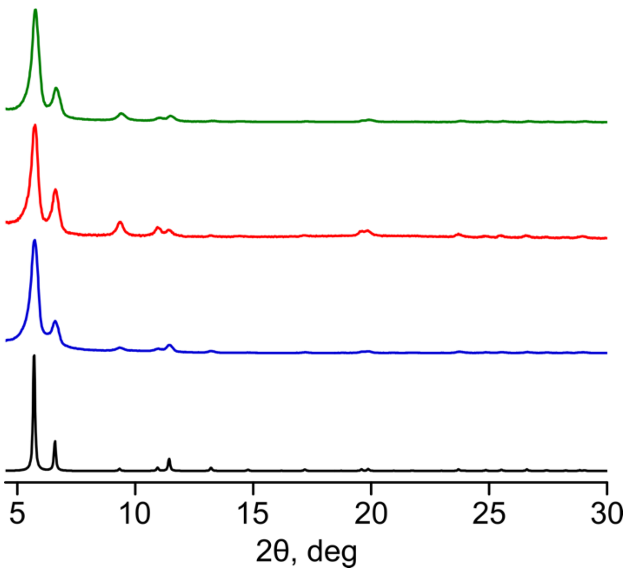


**Supplementary Figure 18**. PXRD patterns of simulated UiO-67 (black),[18] as-synthesized UiO-67 (blue), UiO-67+**2** (red), and UiO-67+**2** after evacuation and irradiation using a 365-nm excitation wavelength (green).

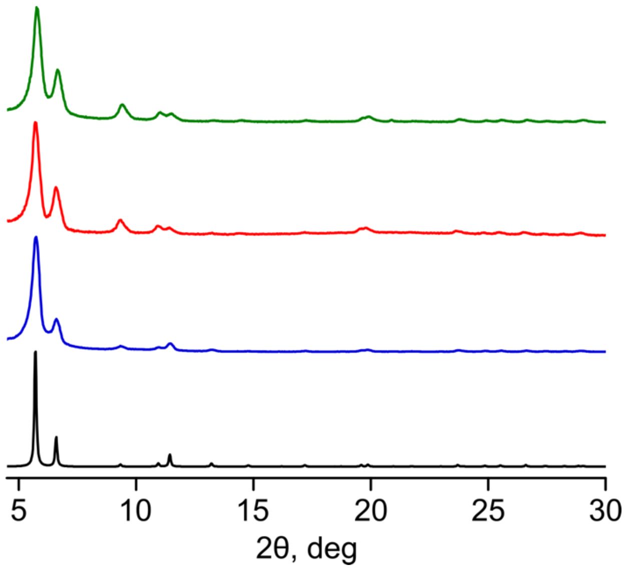


**Supplementary Figure 19**. PXRD patterns of simulated UiO-67 (black),[18] as-synthesized UiO-67 (blue), UiO-67+**5** (red), and UiO-67+**5** after evacuation and irradiation using a 365-nm excitation wavelength (green).

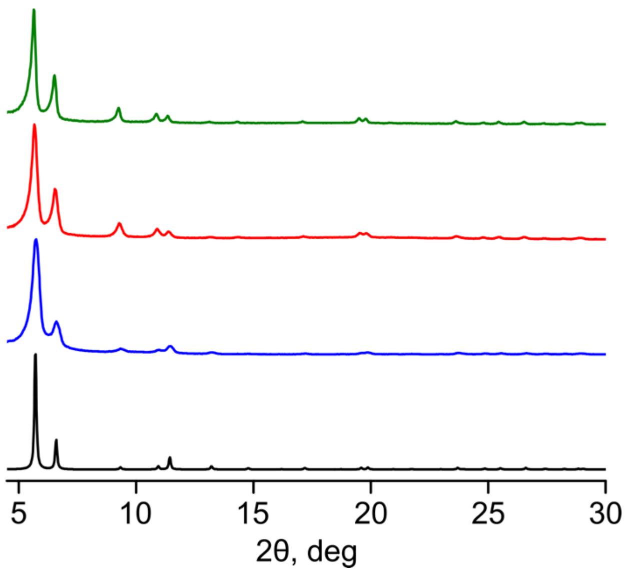


**Supplementary Figure 20**. PXRD patterns of simulated UiO-67 (black),[18] as-synthesized UiO-67 (blue), UiO-67+**6** (red), and UiO-67+**6** after evacuation and irradiation using a 365-nm excitation wavelength (green).

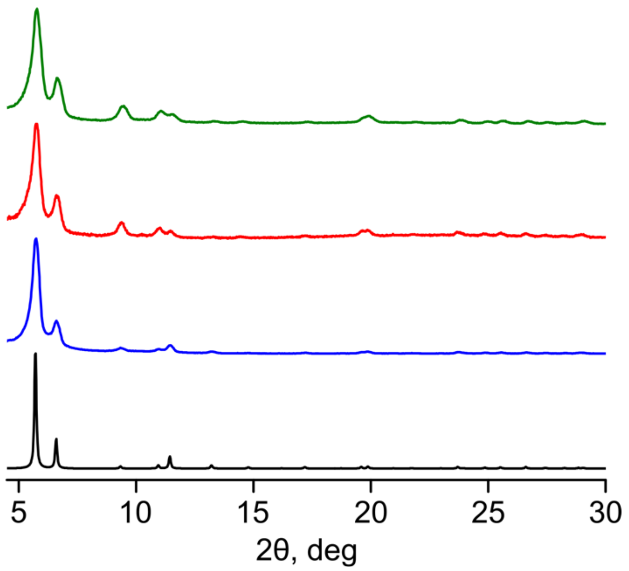


**Supplementary Figure 21**. PXRD patterns of simulated UiO-67 (black),[18] as-synthesized UiO-67 (blue), UiO-67+**11** (red), and UiO-67+**11** after evacuation and irradiation using a 365-nm excitation wavelength (green).

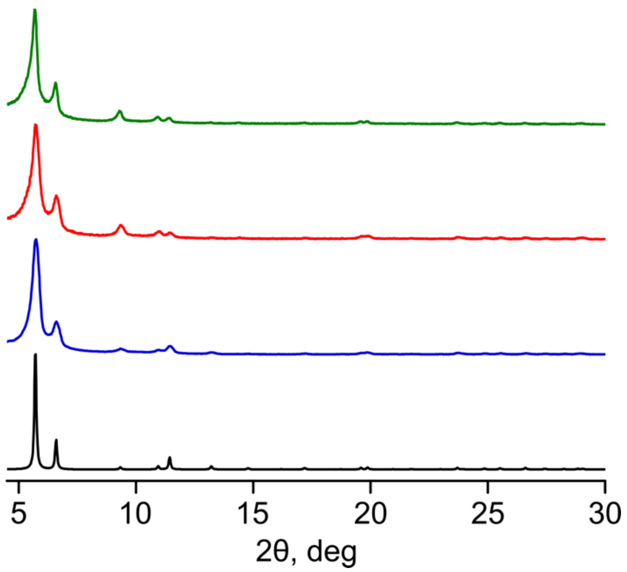


**Supplementary Figure 22**. PXRD patterns of simulated UiO-67 (black),[18] as-synthesized UiO-67 (blue), UiO-67+**2**+**5** (red), and UiO-67+**2**+**5** after evacuation and irradiation using a 365-nm excitation wavelength (green).

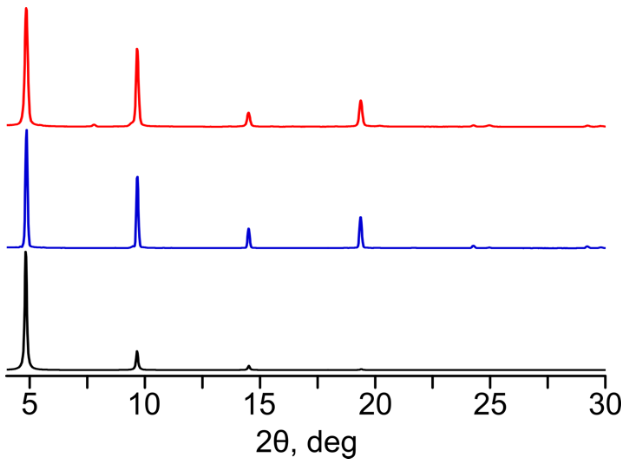


**Supplementary Figure 23**. PXRD patterns of simulated $Zn_2$(**3**)(DBTD) (black),[1] as-synthesized $Zn_2$(**3**)(DBTD) (blue), and $Zn_2$(**3**)(DBTD) after evacuation and irradiation using a 365-nm excitation wavelength (red).

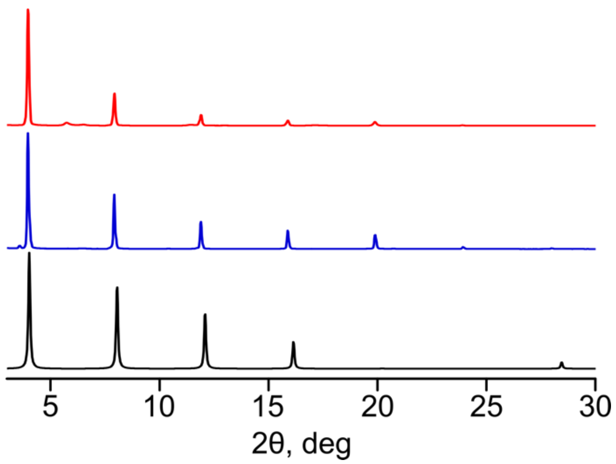


**Supplementary Figure 24**. PXRD patterns of simulated $Zn_2$(**12**)(DBTD) (black),[1] as-synthesized $Zn_2$(**12**)(DBTD) (blue), and $Zn_2$(**12**)(DBTD) after irradiation using a 365-nm excitation wavelength (red).

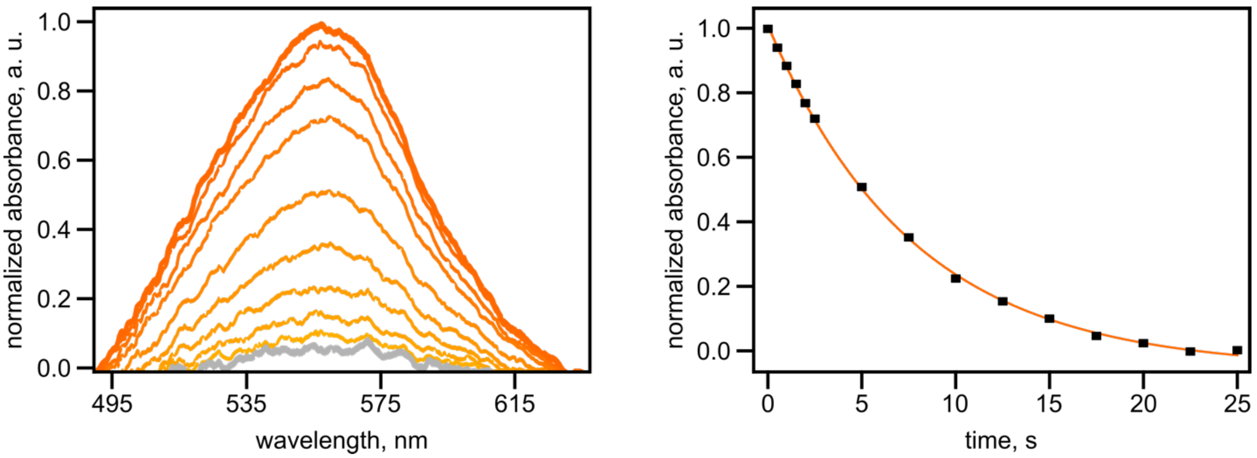


**Supplementary Figure 25**. (*left*) Normalized absorbance spectra of **1** (3 mM in DMF) upon exposure to 365-nm irradiation for 30 s followed by attenuation under visible light ($\lambda_{ex}$ = 400–900 nm). (*right*) Kinetic studies of **1** photoisomerization (3 mM in DMF) showing a decrease in absorbance at 560 nm upon exposure to visible light ($k$ = 0.14 ± 0.02 $s^{-1}$, $R^2$ = 0.99).

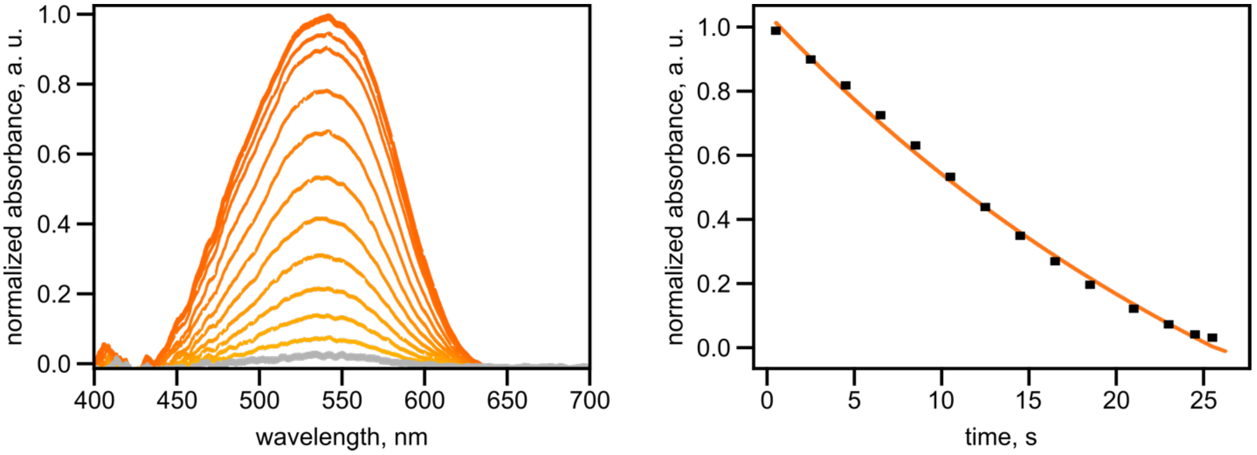


**Supplementary Figure 26**. (*left*) Normalized absorbance spectra of **1** (3 mM in ethanol) upon exposure to 365-nm irradiation for 30 s followed by attenuation under visible light ($\lambda_{ex}$ = 400–900 nm). (*right*) Kinetic studies of **1** photoisomerization (3 mM in ethanol) showing a decrease in absorbance at 550 nm upon exposure to visible light ($k$ = 0.033 ± 0.005 $s^{-1}$, $R^2$ = 0.99).

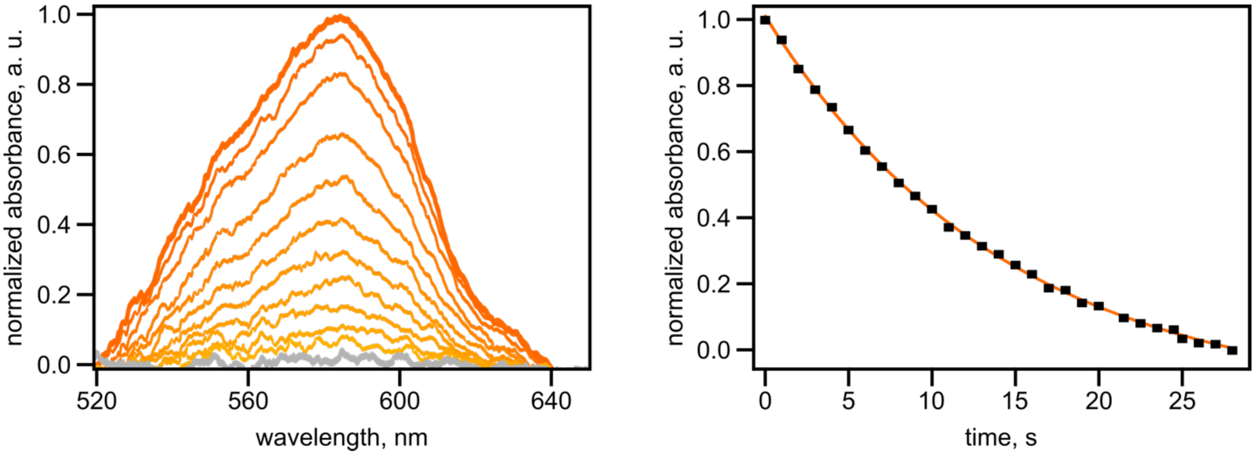


**Supplementary Figure 27**. (*left*) Normalized absorbance spectra of **1** (3 mM in THF) upon exposure to 365-nm irradiation for 30 s followed by attenuation under visible light ($\lambda_{ex}$ = 400–900 nm). (*right*) Kinetic studies of **1** photoisomerization (3 mM in THF) showing a decrease in absorbance at 590 nm upon exposure to visible light ($k$ = 0.074 ± 0.004 $s^{-1}$, $R^2$ = 0.99).

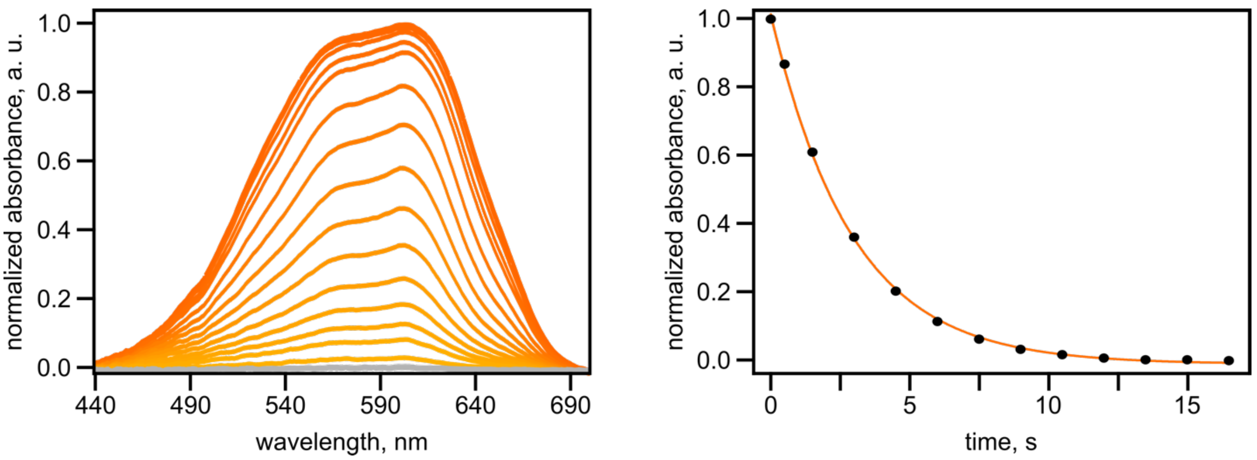


**Supplementary Figure 28**. (*left*) Normalized absorbance spectra of **1** (3 mM in toluene) upon exposure to 365-nm irradiation for 30 s followed by attenuation under visible light ($\lambda_{ex}$ = 400–900 nm). (*right*) Kinetic studies of **1** photoisomerization (3 mM in toluene) showing a decrease in absorbance at 590 nm upon exposure to visible light ($k$ = 0.060 ± 0.007 s$^{-1}$, $R^2$ = 0.99).

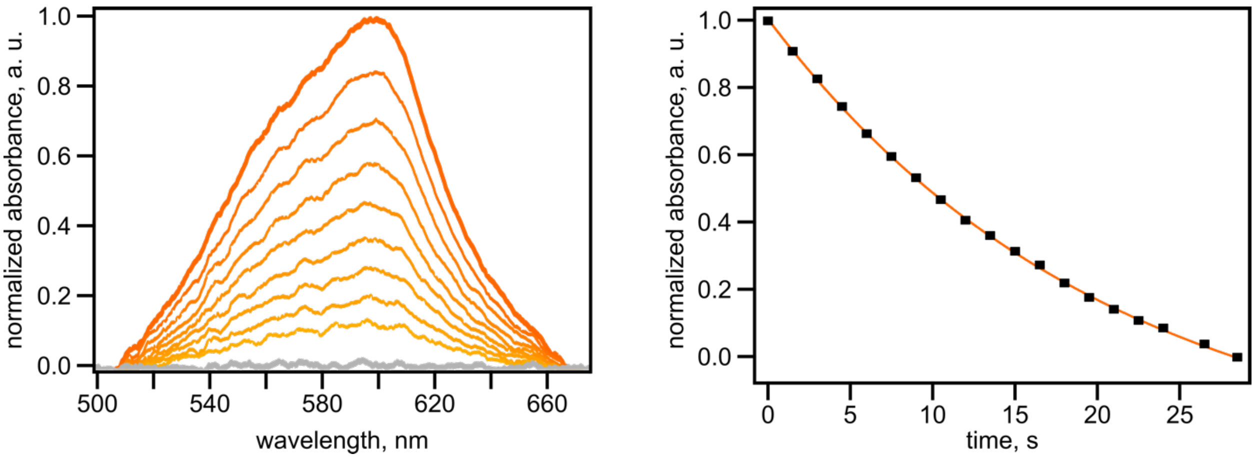


**Supplementary Figure 29**. (*left*) Normalized absorbance spectra of **2** (3 mM in THF) upon exposure to 365-nm irradiation for 30 s followed by attenuation under visible light ($\lambda_{ex}$ = 400–900 nm). (*right*) Kinetic studies of **2** photoisomerization (3 mM in THF) showing a decrease in absorbance at 590 nm upon exposure to visible light ($k$ = 0.048 ± 0.004 s$^{-1}$, $R^2$ = 0.99).

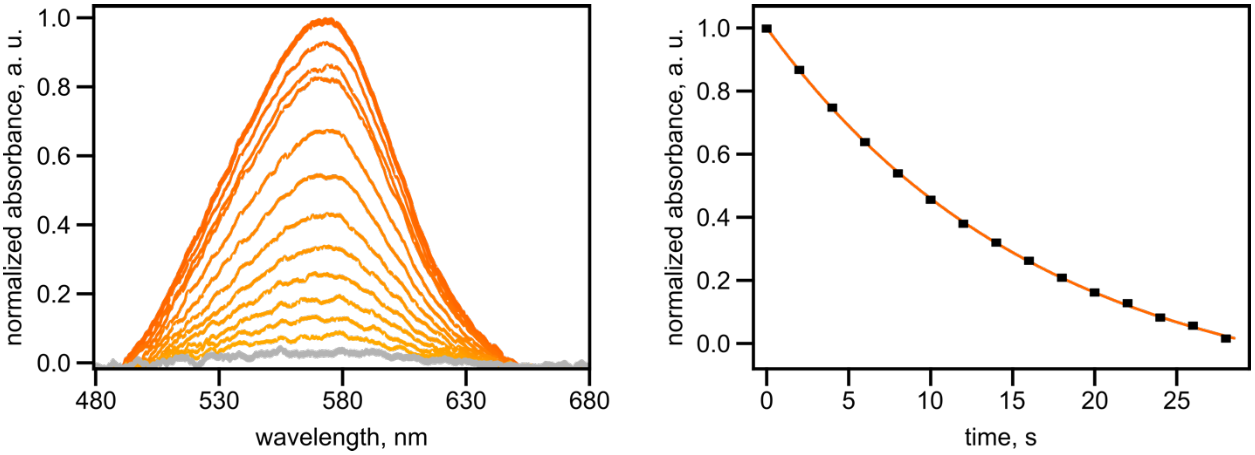


**Supplementary Figure 30**. (*left*) Normalized absorbance spectra of **3** (3 mM in DMF) upon exposure to 365-nm irradiation for 30 s followed by attenuation under visible light ($\lambda_{ex}$ = 400–900 nm). (*right*) Kinetic studies of **3** photoisomerization (3 mM in DMF) showing a decrease in absorbance at 580 nm upon exposure to visible light ($k$ = 0.08 ± 0.01 $s^{-1}$, $R^2$ = 0.99).

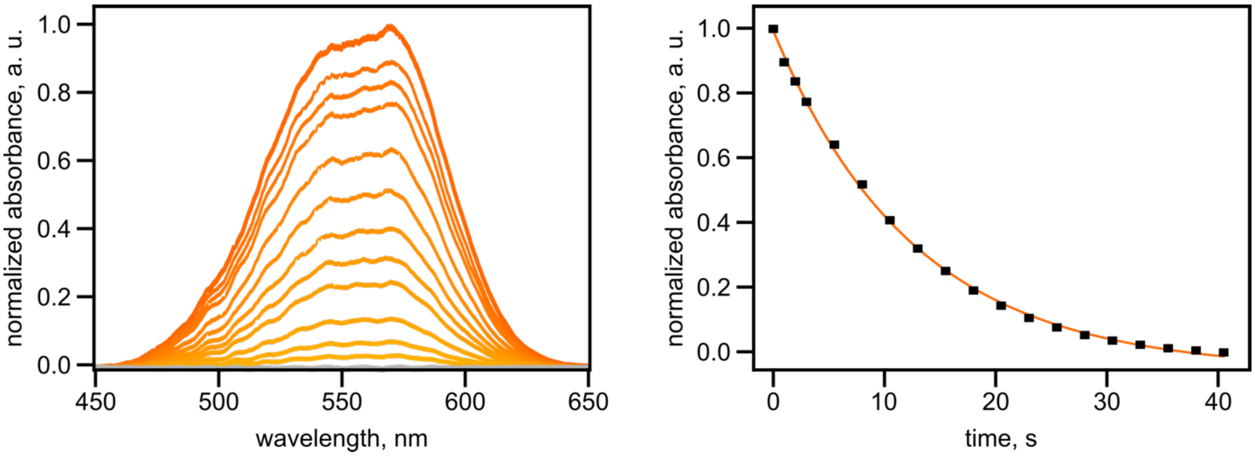


**Supplementary Figure 31**. (*left*) Normalized absorbance spectra of **3** (3 mM in ethanol) upon exposure to 365-nm irradiation for 30 s followed by attenuation under visible light ($\lambda_{ex}$ = 400–900 nm). (*right*) Kinetic studies of **3** photoisomerization (3 mM in ethanol) showing a decrease in absorbance at 590 nm upon exposure to visible light ($k$ = 0.037 ± 0.003 $s^{-1}$, $R^2$ = 0.99).

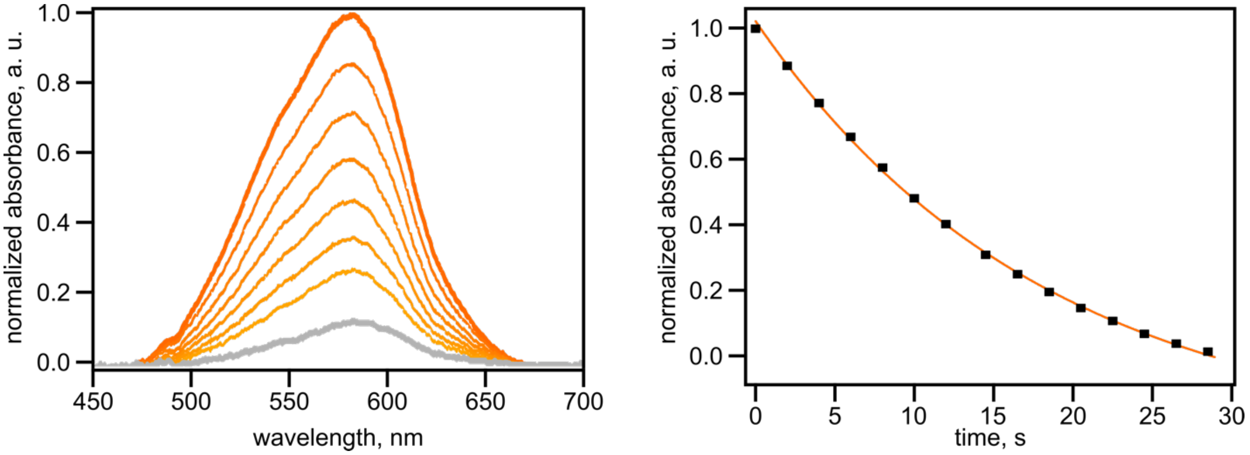


**Supplementary Figure 32**. (*left*) Normalized absorbance spectra of **3** (3 mM in acetonitrile) upon exposure to 365-nm irradiation for 30 s followed by attenuation under visible light ($\lambda_{ex}$ = 400–900 nm). (*right*) Kinetic studies of **3** photoisomerization (3 mM in acetonitrile) showing a decrease in absorbance at 590 nm upon exposure to visible light ($k$ = 0.054 ± 0.004 $s^{-1}$, $R^2$ = 0.99).

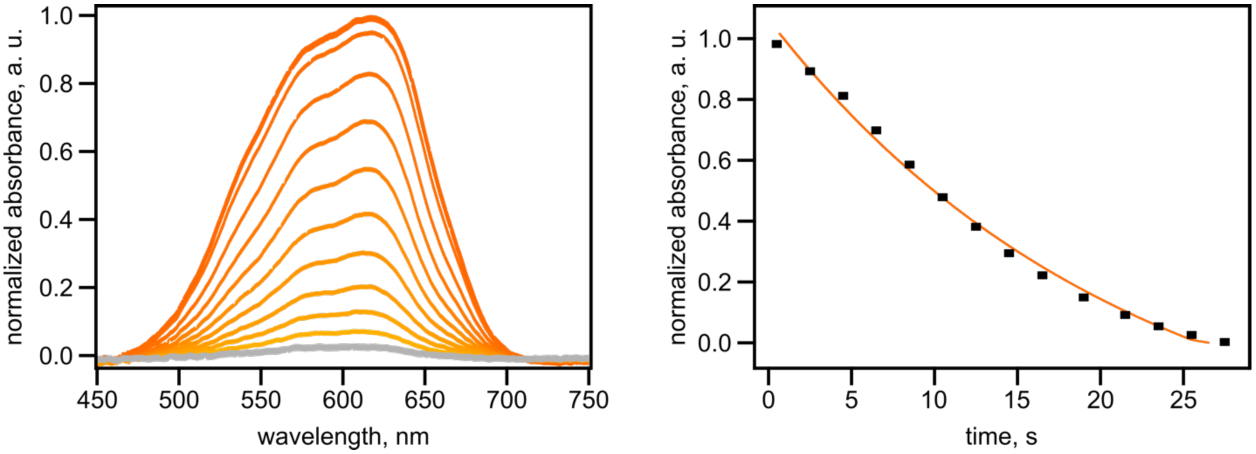


**Supplementary Figure 33**. (*left*) Normalized absorbance spectra of **3** (3 mM in toluene) upon exposure to 365-nm irradiation for 30 s followed by attenuation under visible light ($\lambda_{ex}$ = 400–900 nm). (*right*) Kinetic studies of **3** photoisomerization (3 mM in toluene) showing a decrease in absorbance at 610 nm upon exposure to visible light ($k$ = 0.07 ± 0.02 $s^{-1}$, $R^2$ = 0.99).

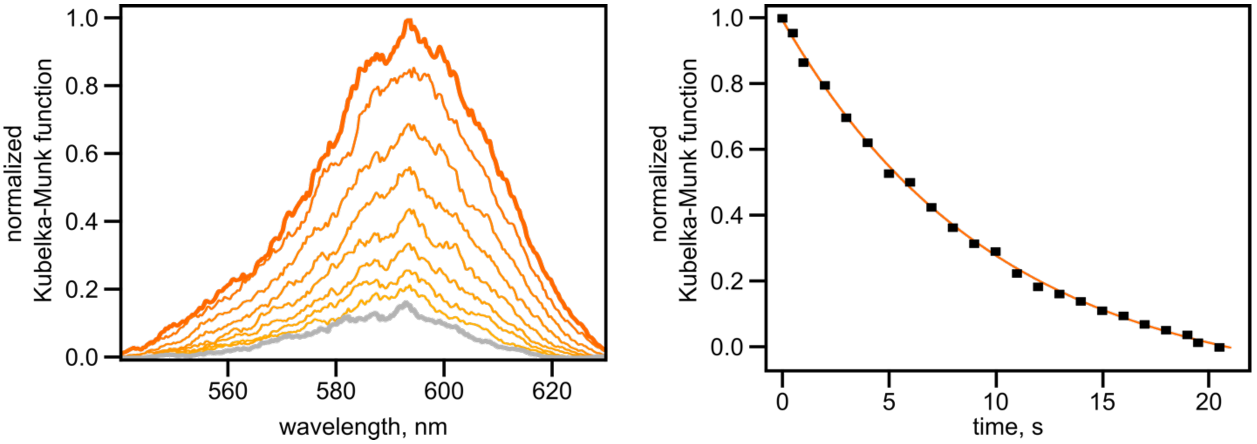


**Supplementary Figure 34**. (*left*) Normalized diffuse reflectance spectra of as-synthesized $Zn_2$(**3**)(DBTD) upon exposure to 365-nm irradiation for 30 s followed by attenuation under visible light ($\lambda_{ex}$ = 400–900 nm). (*right*) Kinetic studies of as-synthesized $Zn_2$(**3**)(DBTD) photoisomerization showing a decrease in absorbance at 590 nm upon exposure to visible light ($k$ = 0.054 ± 0.007 $s^{-1}$, $R^2$ = 0.99).

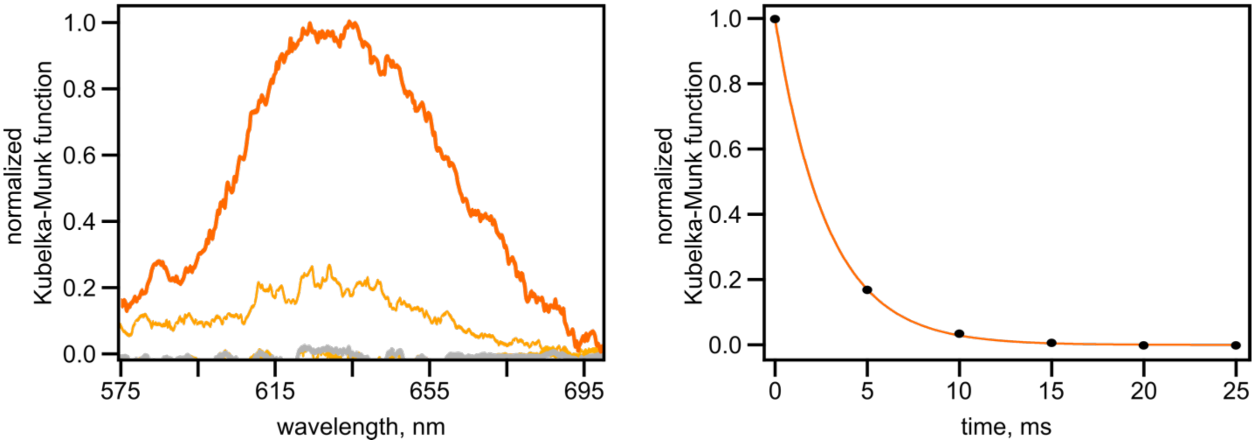


**Supplementary Figure 35**. (*left*) Normalized diffuse reflectance spectra of $Zn_2$(**3**)(DBTD) after evacuation for 24 hours upon exposure to 365-nm irradiation for 30 s followed by attenuation under visible light ($\lambda_{ex}$ = 400–900 nm). (*right*) Kinetic studies of $Zn_2$(**3**)(DBTD) photoisomerization showing a decrease in absorbance at 620 nm upon exposure to visible light ($k$ = 53 ± 2 $s^{-1}$, $R^2$ = 0.99). The presented signal-to-noise data is due to very rapid photoisomerization of **3** within $Zn_2$(**3**)(DBTD).

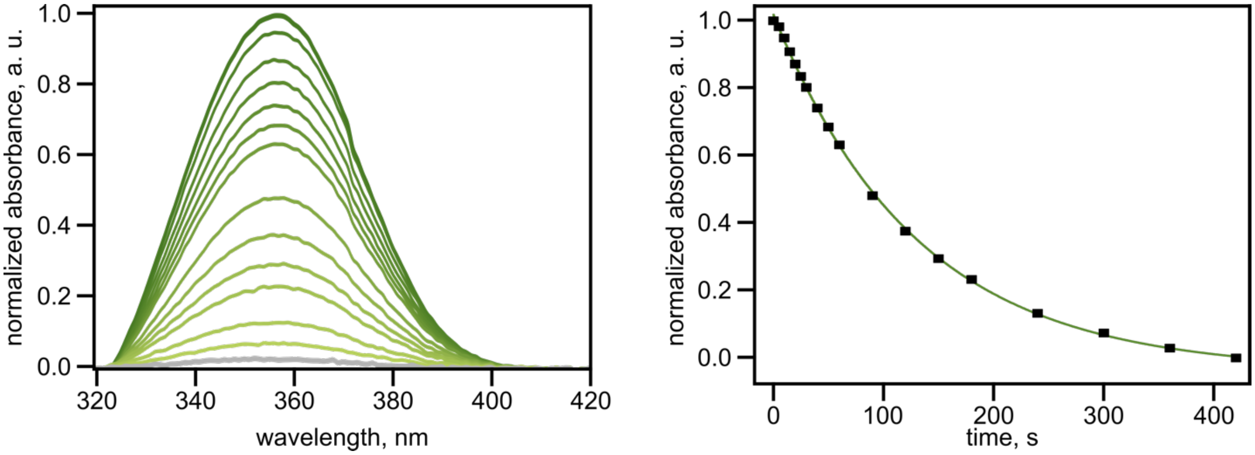


**Supplementary Figure 36**. (*left*) Normalized absorbance spectra of **4** (30 µM in DMF) upon exposure to 365-nm irradiation. *E*-to-*Z* photoisomerization under 365-nm irradiation is shown by the green-to-gray gradient. (*right*) Kinetic studies of **4** photoisomerization showing a decrease in absorbance at 360 nm upon exposure to visible light ($k$ = 0.0074 ± 0.0003 $s^{-1}$, $R^2$ = 0.99).

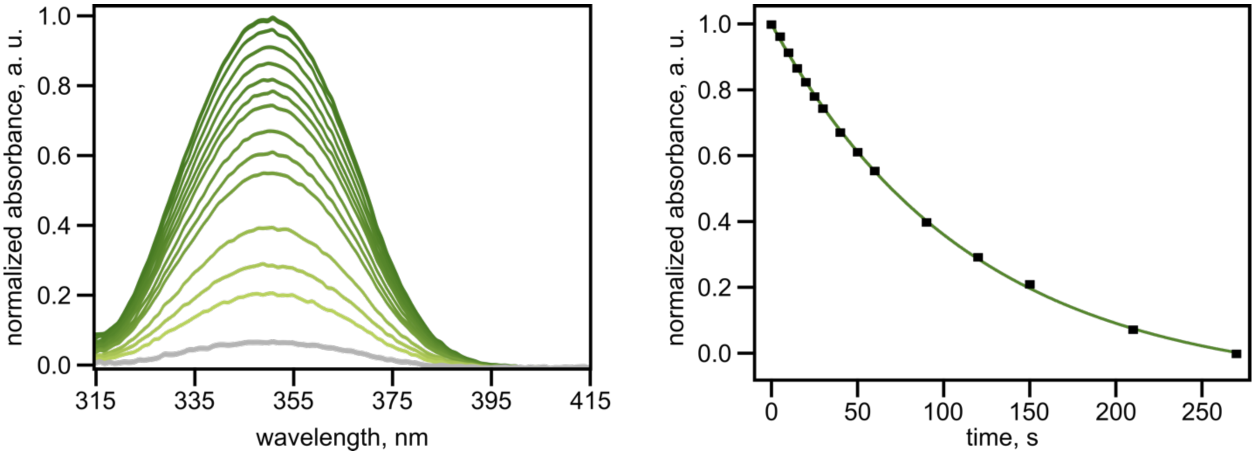


**Supplementary Figure 37**. (*left*) Normalized absorbance spectra of **4** (30 µM in toluene) upon exposure to 365-nm irradiation. *E*-to-*Z* photoisomerization under 365-nm irradiation is shown by the green-to-gray gradient. (*right*) Kinetic studies of **4** photoisomerization showing a decrease in absorbance at 350 nm upon exposure to UV light ($k$ = 0.0086 ± 0.0003 $s^{-1}$, $R^2$ = 0.99).

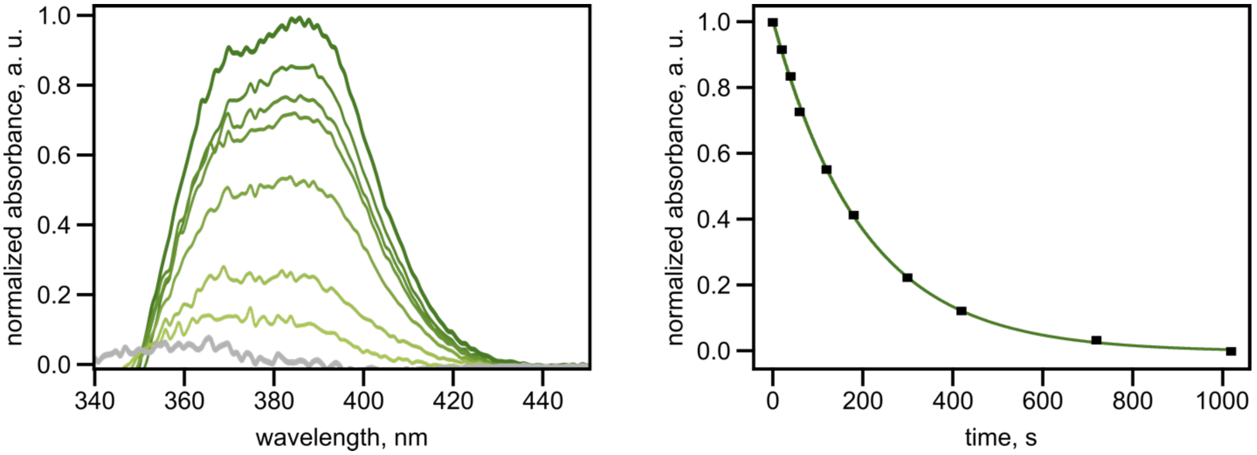


**Supplementary Figure 38**. (*left*) Normalized absorbance spectra of **5** (30 µM in DMF) upon exposure to 365-nm irradiation. *E*-to-*Z* photoisomerization under 365-nm irradiation is shown by the green-to-gray gradient. (*right*) Kinetic studies of **5** photoisomerization showing a decrease in absorbance at 390 nm upon exposure to visible light ($k$ = 0.00484 ± 0.00001 $s^{-1}$, $R^2$ = 0.99).

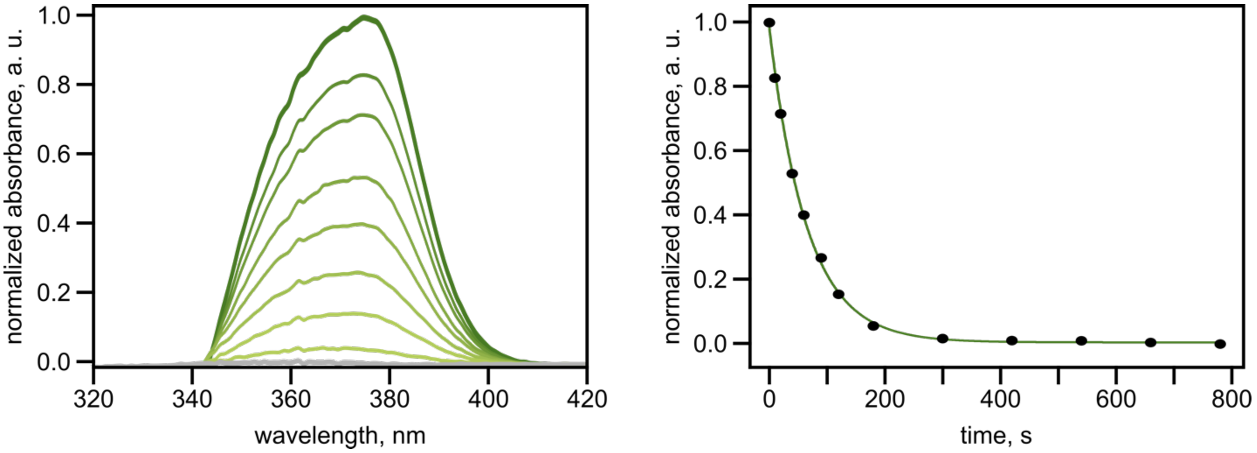


**Supplementary Figure 39**. (*left*) Normalized absorbance spectra of **5** (30 µM in ethanol) upon exposure to 365-nm irradiation. *E*-to-*Z* photoisomerization under 365-nm irradiation is shown by the green-to-gray gradient. (*right*) Kinetic studies of **5** photoisomerization showing a decrease in absorbance at 370 nm upon exposure to visible light ($k$ = 0.016 ± 0.003 $s^{-1}$, $R^2$ = 0.99).

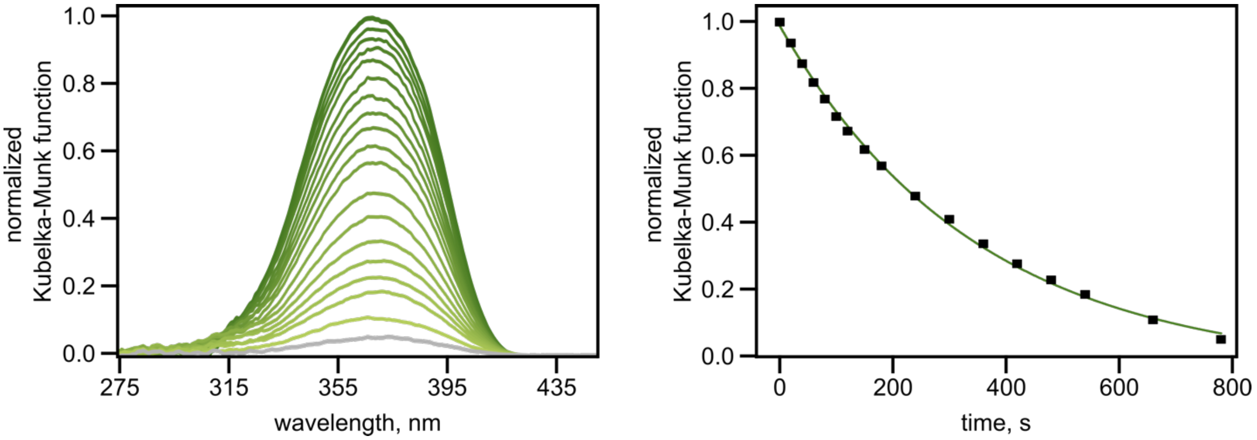


**Supplementary Figure 40**. (*left*) Normalized diffuse reflectance spectra of as-synthesized UiO-67+**5** upon exposure to 365-nm irradiation. *E*-to-*Z* photoisomerization under 365-nm irradiation is shown by the green-to-gray gradient. (*right*) Kinetic studies of as-synthesized UiO-67+**5** photoisomerization showing a decrease in absorbance at 360 nm upon exposure to visible light ($k$ = 0.005 ± 0.002 $s^{-1}$, $R^2$ = 0.99).

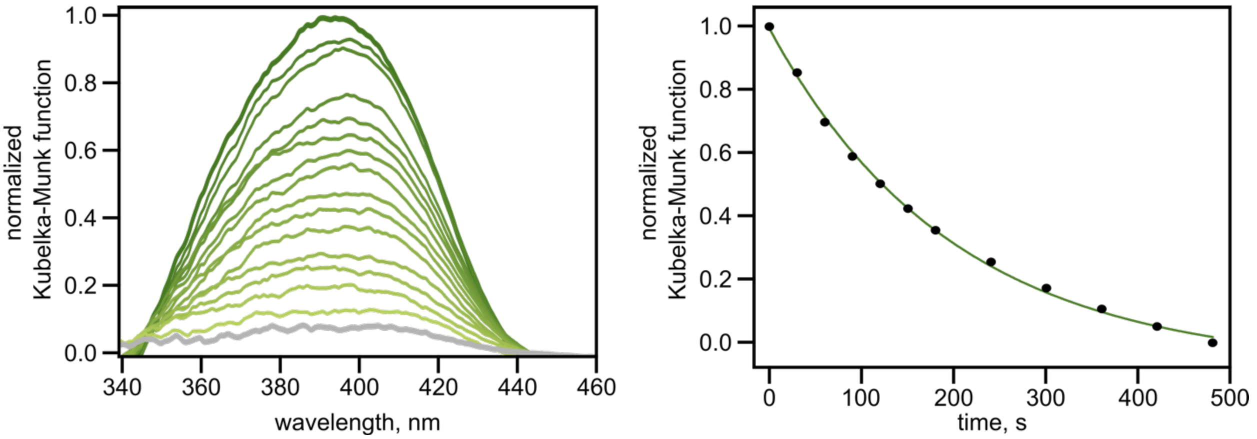


**Supplementary Figure 41**. (*left*) Normalized diffuse reflectance spectra of UiO-67+**5** after evacuation for 24 h and exposure to 365-nm irradiation. *E*-to-*Z* photoisomerization under 365-nm irradiation is shown by the green-to-gray gradient. (*right*) Kinetic studies of UiO-67+**5** photoisomerization showing a decrease in absorbance at 390 nm upon exposure to UV light ($k$ = 0.013 ± 0.004 $s^{-1}$, $R^2$ = 0.99).

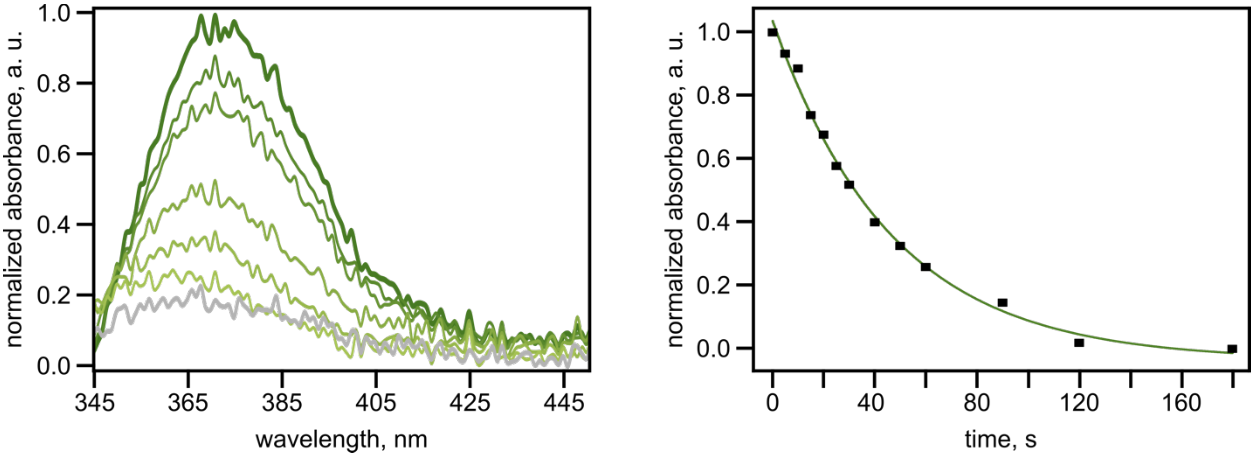


**Supplementary Figure 42**. (*left*) Normalized absorbance spectra of **6** (30 μM in DMF) upon exposure to 365-nm irradiation. *Z*-to-*E* photoisomerization under 365-nm irradiation is shown by the green-to-gray gradient. (*right*) Kinetic studies of **6** photoisomerization showing a decrease in absorbance at 370 nm upon exposure to UV light ($k$ = 0.015 ± 0.005 $s^{-1}$, $R^2$ = 0.98).

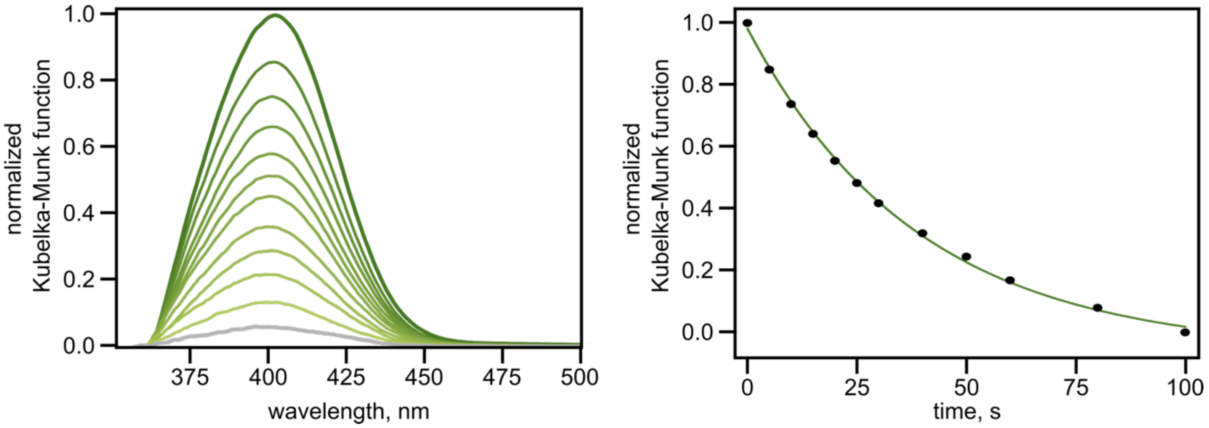


**Supplementary Figure 43**. (*left*) Normalized diffuse reflectance spectra of as-synthesized UiO-67+**6** upon exposure to 365-nm irradiation. *Z*-to-*E* photoisomerization under 365-nm irradiation is shown by the green-to-gray gradient. (*right*) Kinetic studies of as-synthesized UiO-67+**6** photoisomerization showing an increase in absorbance at 405 nm upon exposure to UV light ($k$ = 0.029 ± 0.003 $s^{-1}$, $R^2$ = 0.99).

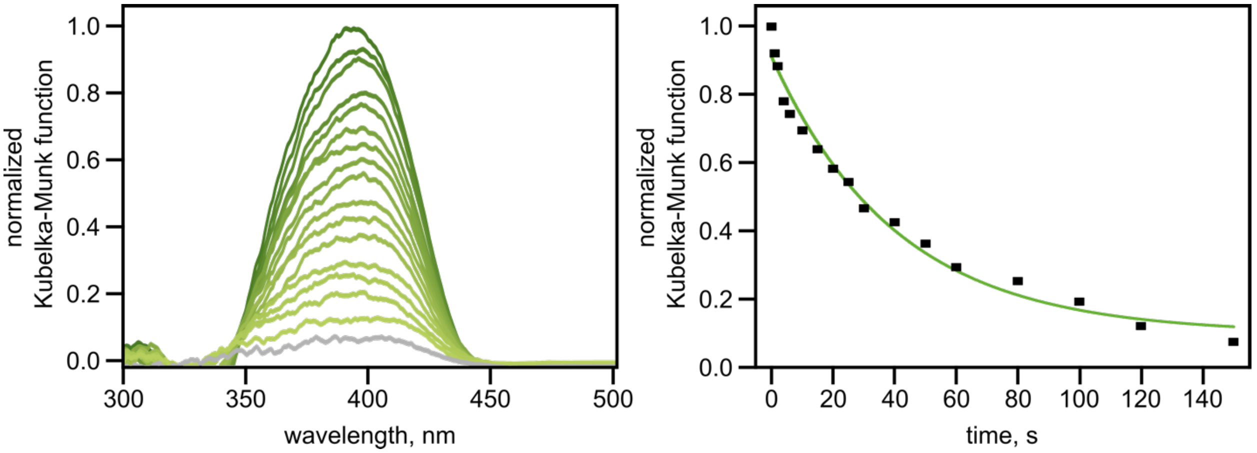


**Supplementary Figure 44**. (*left*) Normalized diffuse reflectance spectra of UiO-67+**6** after evacuation for 24 h and exposure to 365-nm irradiation. *Z*-to-*E* photoisomerization under 365-nm irradiation is shown by the green-to-gray gradient. (*right*) Kinetic studies of UiO-67+**6** photoisomerization showing a decrease in absorbance at 405 nm upon exposure to UV light ($k$ = 0.026 ± 0.002 $s^{-1}$, $R^2$ = 0.98).

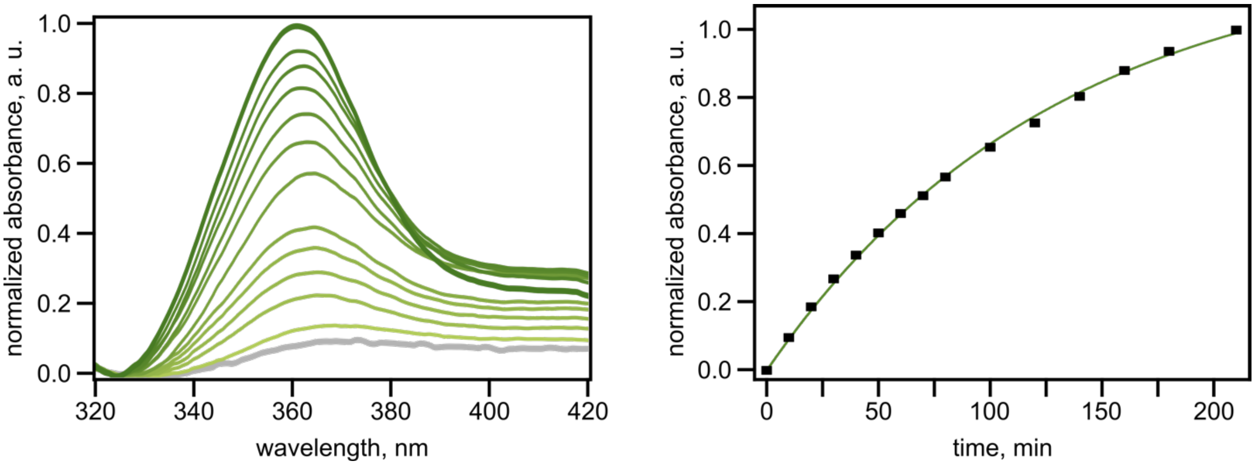


**Supplementary Figure 45**. (*left*) Normalized absorbance spectra of **7** (10 μM in methanol) upon exposure to 365-nm irradiation. *E*-to-*Z* photoisomerization under 365-nm irradiation is shown by the green-to-gray gradient. (*right*) Kinetic studies of **7** photoisomerization showing a decrease in absorbance at 360 nm upon exposure to visible light ($k$ = 6.0 ± 0.7 × $10^{-4}$ $s^{-1}$, $R^2$ = 0.99).

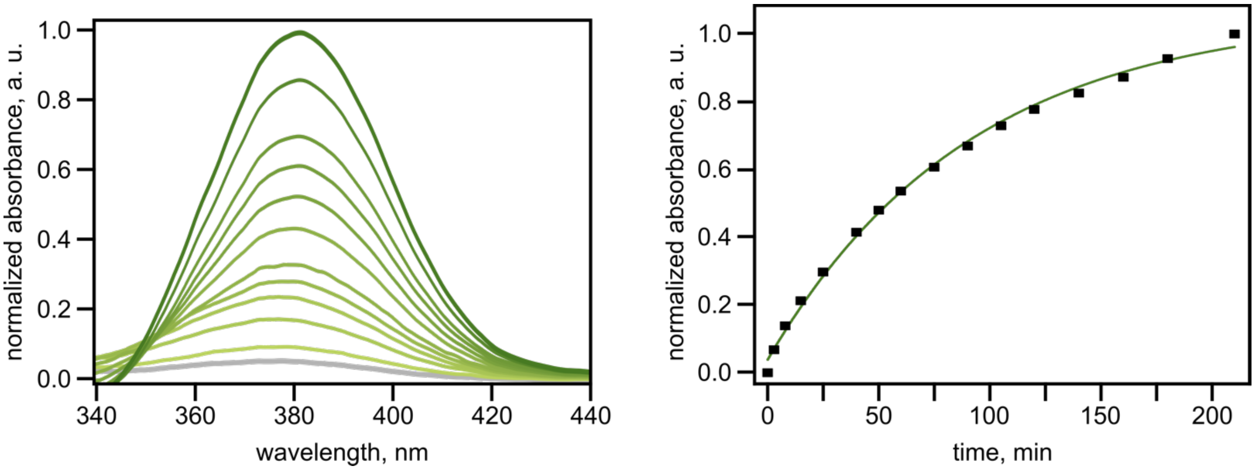


**Supplementary Figure 46**. (*left*) Normalized absorbance spectra of **8** (10 μM in methanol) upon exposure to 365-nm irradiation. *E*-to-*Z* photoisomerization under 365-nm irradiation is shown by the green-to-gray gradient. (*right*) Kinetic studies of **8** photoisomerization showing a decrease in absorbance at 380 nm upon exposure to visible light ($k = 6.0 \pm 0.8 \times 10^{-4}$ $s^{-1}$, $R^2 = 0.99$).

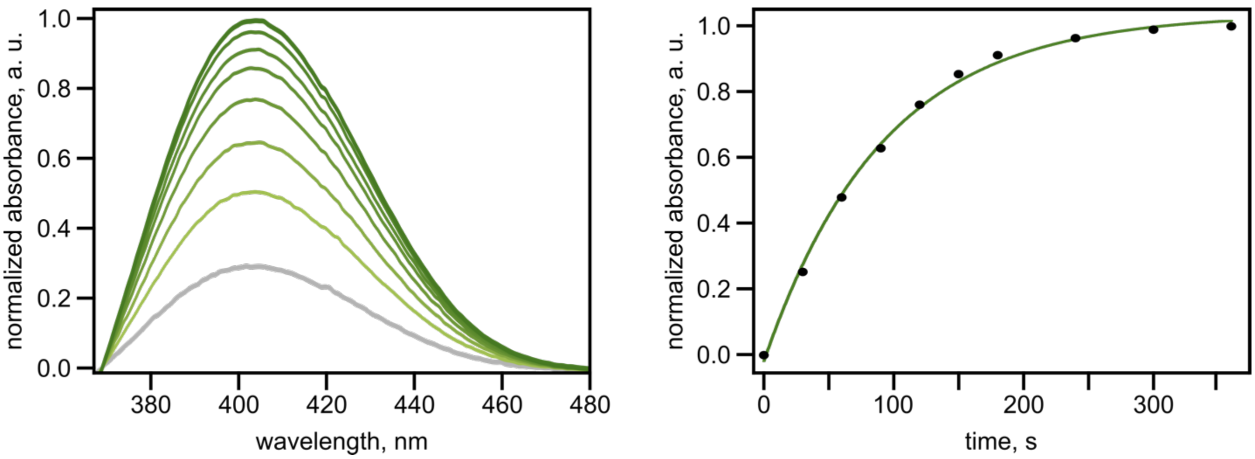


**Supplementary Figure 47**. (*left*) Normalized absorbance spectra of **9** (10 μM in toluene) upon exposure to 365-nm irradiation. *E*-to-*Z* photoisomerization under 365-nm irradiation is shown by the green-to-gray gradient. (*right*) Kinetic studies of **9** photoisomerization (10 μM in toluene) showing an increase in absorbance at 405 nm upon exposure to UV light ($k = 0.03 \pm 0.01$ $s^{-1}$, $R^2 = 0.99$).

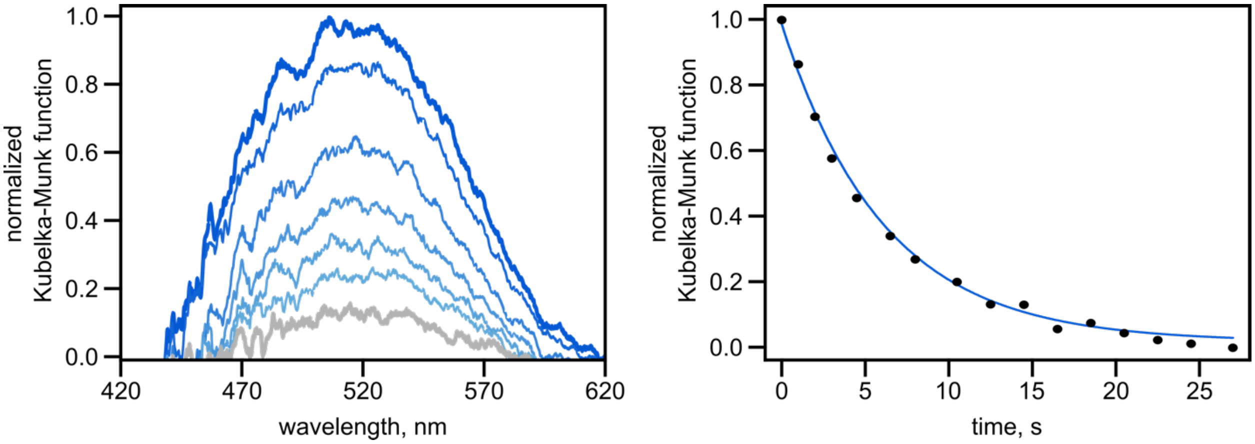


**Supplementary Figure 48**. (*left*) Normalized diffuse reflectance spectra of **10** (solid state) upon exposure to 365-nm irradiation for 30s followed by attenuation under visible light ($\lambda_{ex}$ = 400–900 nm). (*right*) Kinetic studies of **10** photoisomerization (solid state) showing a decrease in absorbance at 520 nm upon exposure to visible light ($k$ = 0.13 ± 0.03 $s^{-1}$, $R^2$ = 0.99).

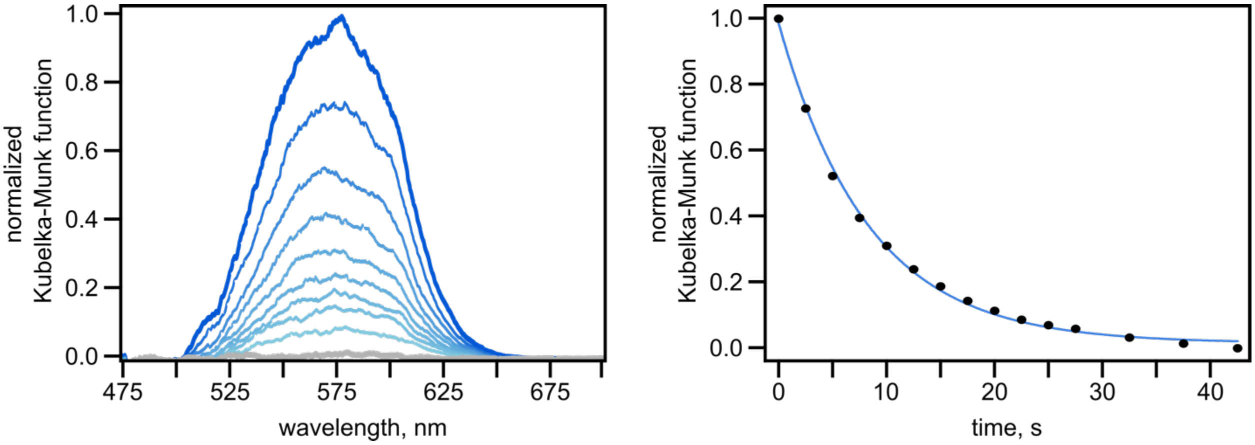


**Supplementary Figure 49**. (*left*) Normalized diffuse reflectance spectra of **11** (solid state) upon exposure to 365-nm irradiation for 30s followed by attenuation under visible light ($\lambda_{ex}$ = 400–900 nm). (*right*) Kinetic studies of **11** photoisomerization (solid state) showing a decrease in absorbance at 575 nm upon exposure to visible light ($k$ = 0.0337 ± 0.0005 $s^{-1}$, $R^2$ = 0.99).

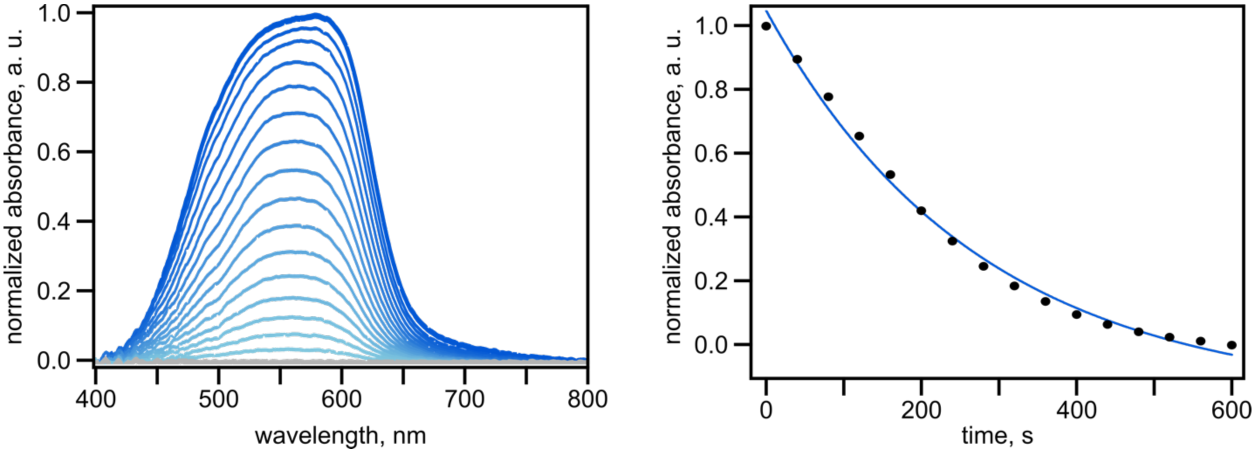


**Supplementary Figure 50**. (*left*) Normalized absorbance spectra of **11** (3 mM in DMF) upon exposure to 365-nm irradiation for 30s followed by attenuation under visible light ($\lambda_{ex}$ = 400–900 nm). (*right*) Kinetic studies of **11** photoisomerization (3 mM in DMF) showing a decrease in absorbance at 570 nm upon exposure to visible light ($k$ = 0.19 ± 0.01 $s^{-1}$, $R^2$ = 0.99).

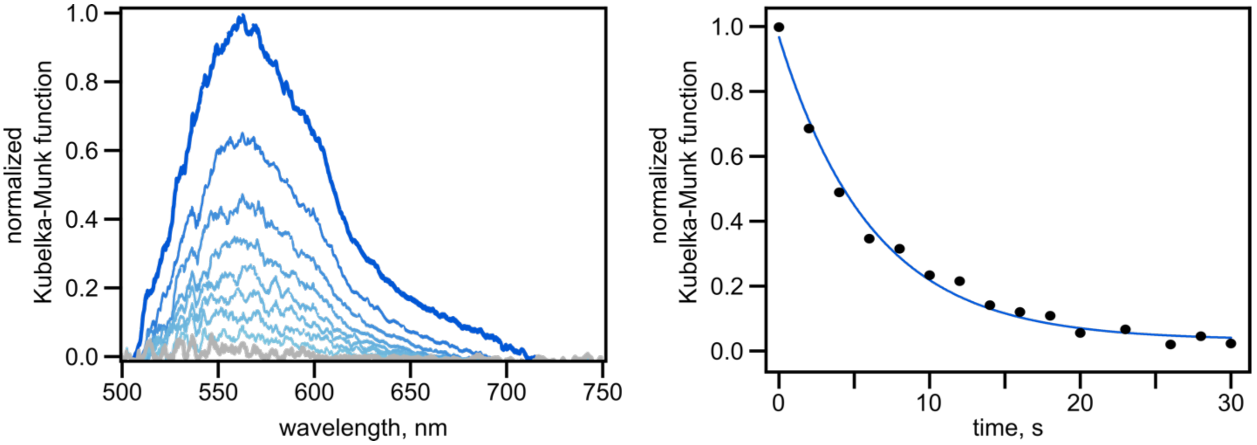


**Supplementary Figure 51**. (*left*) Normalized diffuse reflectance spectra of as-synthesized UiO-67+**11** upon exposure to 365-nm irradiation for 30s followed by attenuation under visible light ($\lambda_{ex}$ = 400–900 nm). (*right*) Kinetic studies of as-synthesized UiO-67+**11** photoisomerization (solid state) showing a decrease in absorbance at 570 nm upon exposure to visible light ($k$ = 0.32 ± 0.02 $s^{-1}$, $R^2$ = 0.98).

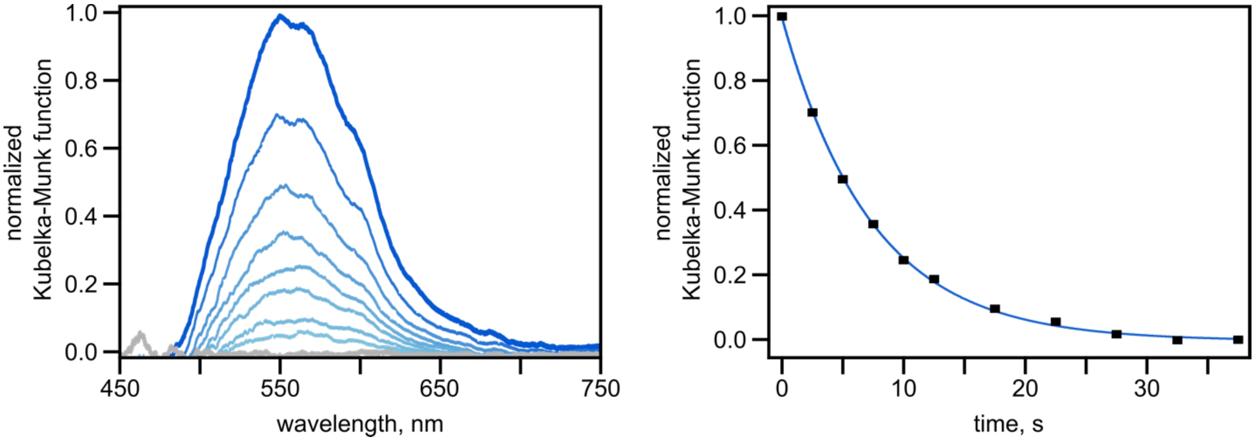


**Supplementary Figure 52**. (*left*) Normalized diffuse reflectance spectra of UiO-67+**11** after evacuation for 24 hours upon exposure to 365-nm irradiation for 30 s followed by attenuation under visible light ($\lambda_{ex}$ = 400–900 nm). (*right*) Kinetic studies of evacuated UiO-67+**11** photoisomerization showing a decrease in absorbance at 560 nm upon exposure to visible light ($k$ = 0.177 ± 0.003 $s^{-1}$, $R^2$ = 0.99).

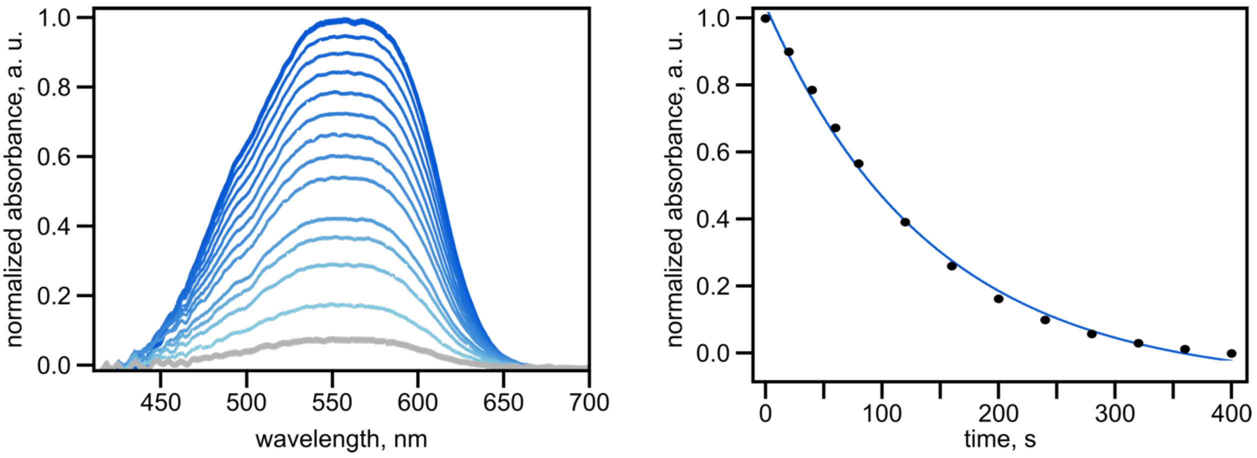


**Supplementary Figure 53**. (*left*) Normalized absorbance spectra of **12** (3 mM in DMF) upon exposure to 365-nm irradiation for 30s followed by attenuation under visible light ($\lambda_{ex}$ = 400–900 nm). (*right*) Kinetic studies of **12** photoisomerization (3 mM in DMF) showing a decrease in absorbance at 550 nm upon exposure to visible light ($k$ = 0.037 ± 0.003 $s^{-1}$, $R^2$ = 0.99).

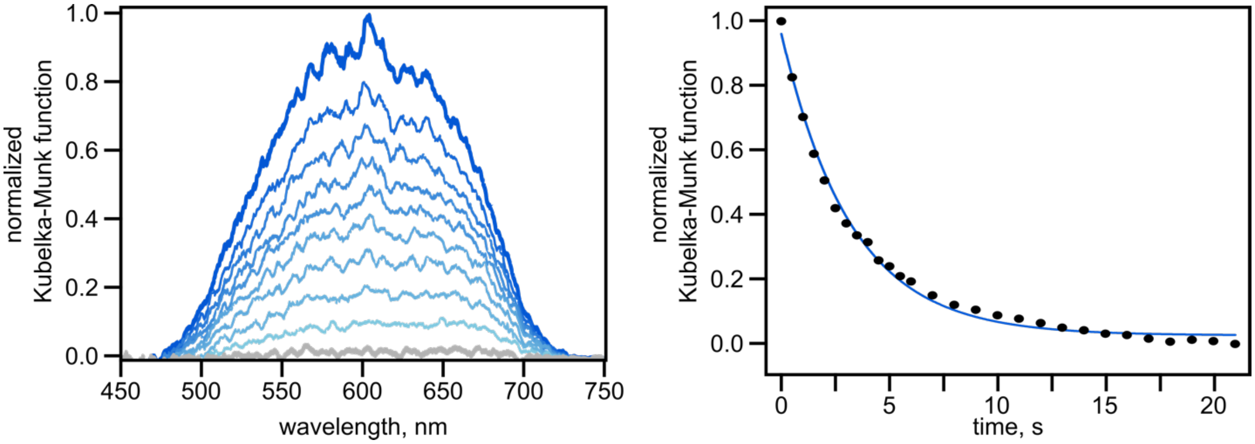


**Supplementary Figure 54**. (*left*) Normalized diffuse reflectance spectra of **12** (solid state) upon exposure to 365-nm irradiation for 30s followed by attenuation under visible light ($\lambda_{ex}$ = 400–900 nm). (*right*) Kinetic studies of **12** photoisomerization (solid state) showing a decrease in absorbance at 600 nm upon exposure to visible light ($k$ = 0.36 ± 0.06 $s^{-1}$, $R^2$ = 0.99).

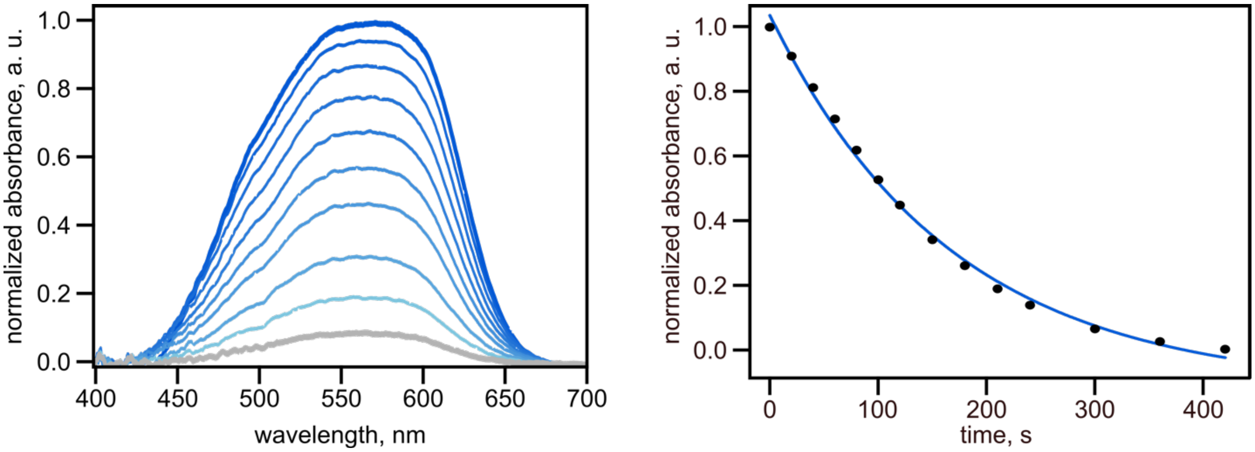


**Supplementary Figure 55**. (*left*) Normalized absorbance spectra of **12** (3 mM in ethanol) upon exposure to 365-nm irradiation for 30s followed by attenuation under visible light ($\lambda_{ex}$ = 400–900 nm). (*right*) Kinetic studies of **12** photoisomerization (3 mM in ethanol) showing a decrease in absorbance at 550 nm upon exposure to visible light ($k$ = 0.005 ± 0.001 $s^{-1}$, $R^2$ = 0.99).

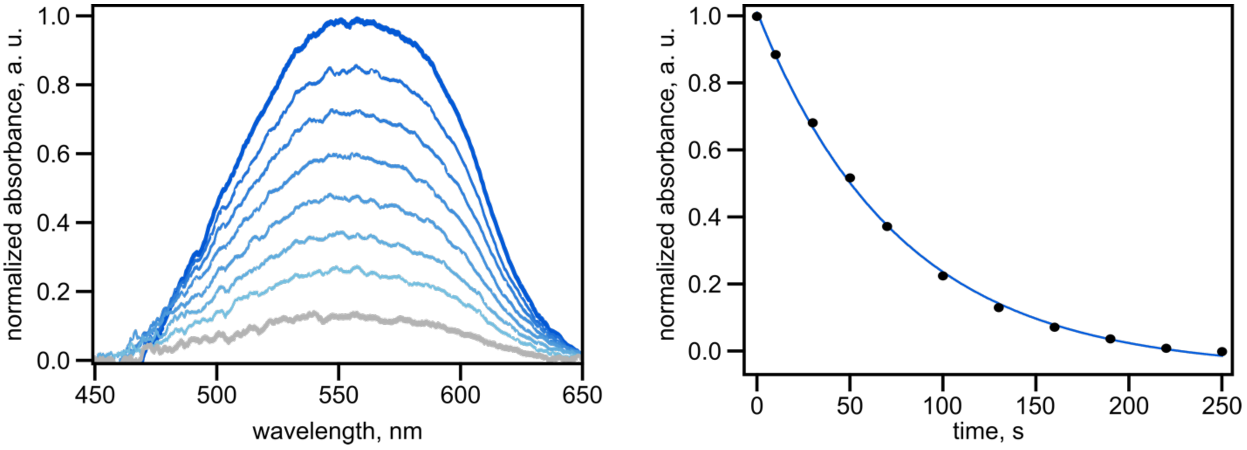


**Supplementary Figure 56**. (*left*) Normalized absorbance spectra of **12** (3 mM in toluene) upon exposure to 365-nm irradiation for 30s followed by attenuation under visible light ($\lambda_{ex}$ = 400–900 nm). (*right*) Kinetic studies of **12** photoisomerization (3 mM in toluene) showing a decrease in absorbance at 550 nm upon exposure to visible light ($k$ = 0.009 ± 0.001 $s^{-1}$, $R^2$ = 0.99).

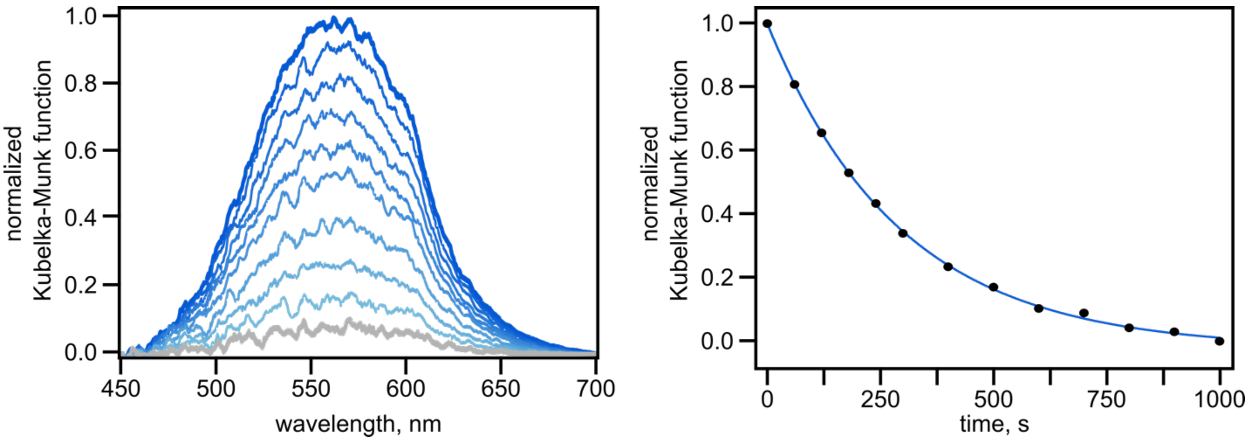


**Supplementary Figure 57**. (*left*) Normalized diffuse reflectance spectra of as-synthesized $Zn_2$(**12**)(DBTD) upon exposure to 365-nm irradiation for 30 s followed by attenuation under visible light ($\lambda_{ex}$ = 400–900 nm). (*right*) Kinetic studies of as-synthesized $Zn_2$(**12**)(DBTD) photoisomerization showing a decrease in absorbance at 560 nm upon exposure to visible light ($k$ = 0.0033 ± 0.0003 $s^{-1}$, $R^2$ = 0.99).

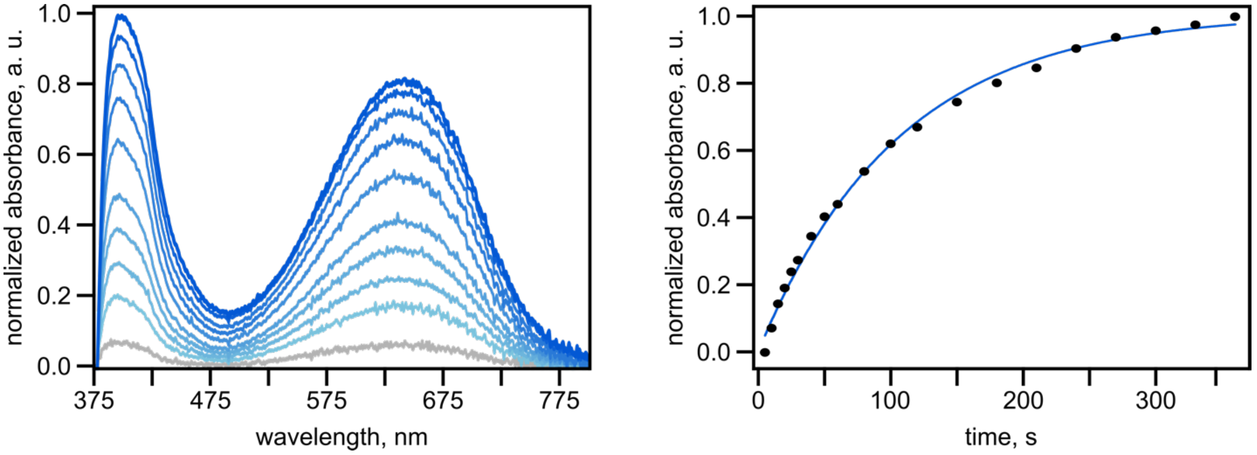


**Supplementary Figure 58**. (*left*) Normalized absorbance spectra of **13** (10 μM in acetonitrile) upon exposure to 365-nm irradiation for 400 s. (*right*) Kinetic studies of **13** photoisomerization (10 μM in acetonitrile) showing an increase in absorbance at 380 nm upon exposure to UV light ($k$ = 0.010 ± 0.0006 $s^{-1}$, $R^2$ = 0.99).

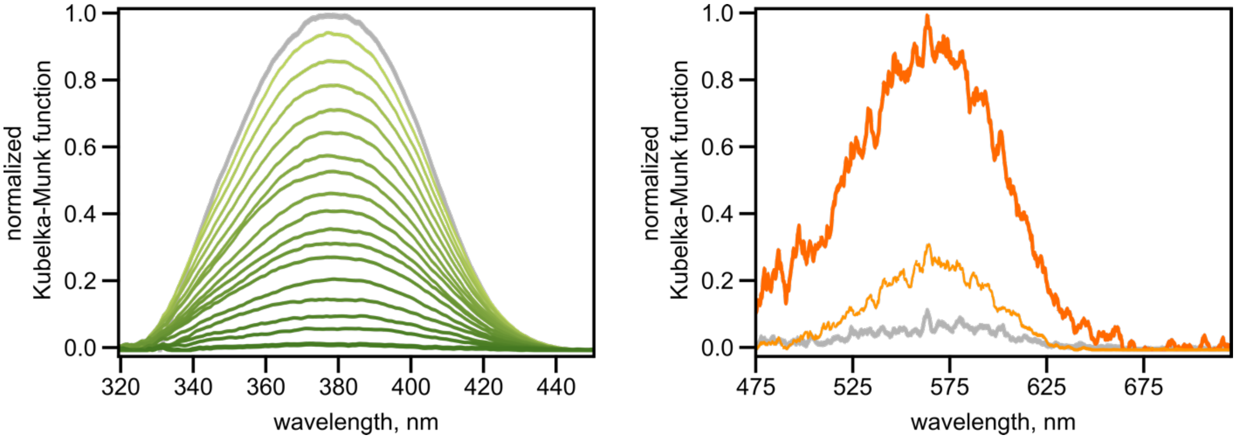


**Supplementary Figure 59**. Normalized diffuse reflectance spectra of UiO-67+**2**+**5** after evacuation for 48 hours. (*left*) Changes in the diffuse reflectance profile corresponding to **5** upon exposure to a 365-nm excitation wavelength. (*right*) Changes in the diffuse reflectance profile corresponding to **2** upon exposure to a 365-nm excitation wavelength for 30 s followed by exposure to visible light ($\lambda_{ex}$ = 400–900 nm).

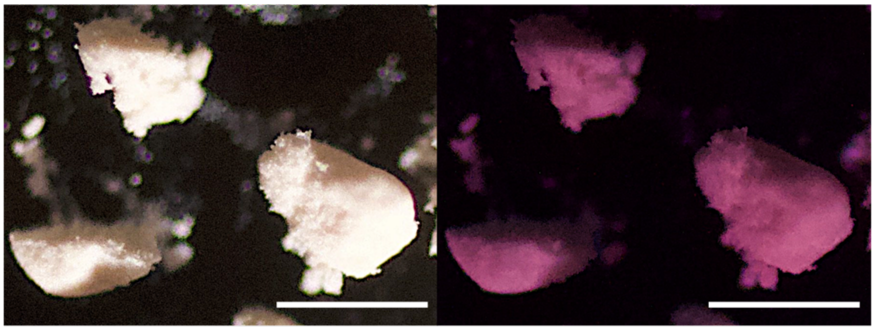

**Supplementary Figure 60**. Epifluorescence microscopic images of UiO-67+**2**+**5** before (*left*) and after (*right*) exposure to a 365-nm excitation wavelength for 30 s, demonstrating the presence of **2**, which is emissive. The scale bar represents 55 μm. Presence of **2** and **5** were also confirmed by $^1$H NMR spectroscopic analysis on the digested MOF samples (Supplementary Fig. 17).

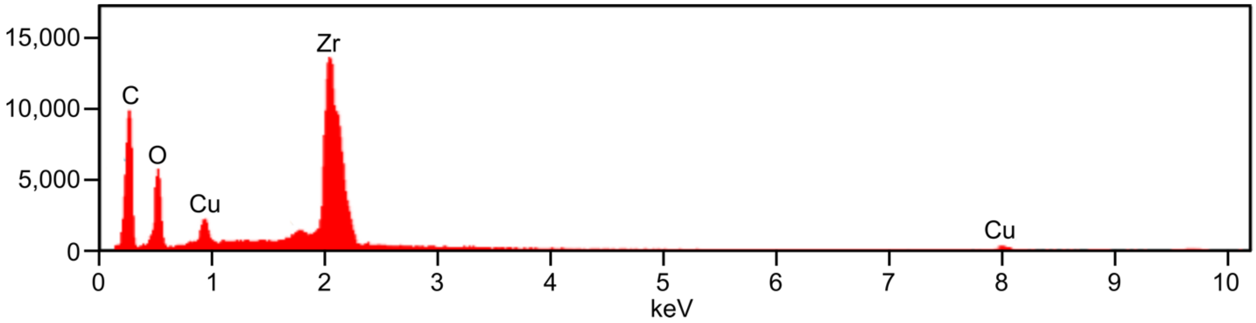


**Supplementary Figure 61**. EDX spectrum of UiO-67+**2**+**5** after exposure to a copper solution to promote binding of copper cations to merocyanine and the defect sites of the UiO-67 host matrix.

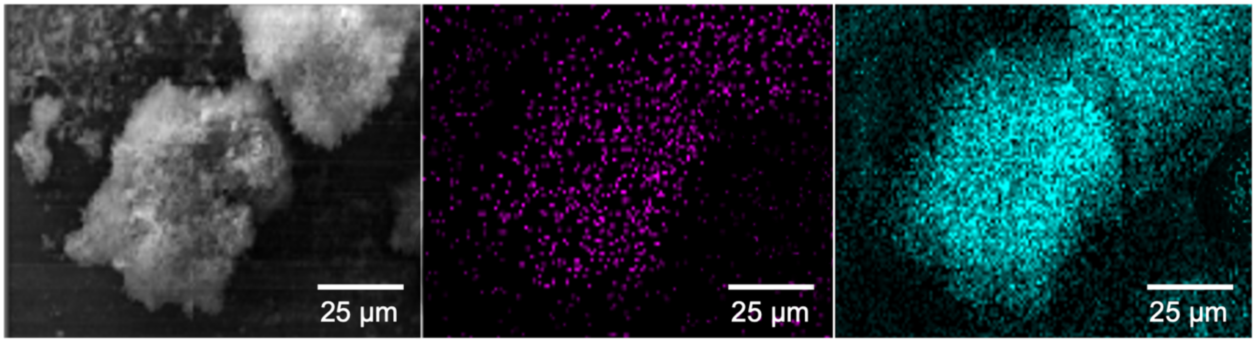


**Supplementary Figure 62**. Scanning electron micrographs of UiO-67+**2**+**5** after coordination of copper cations by merocyanine and the defect sites of the UiO-67 host matrix. Presence of copper is shown in pink, while the zirconium metal nodes are shown in blue.

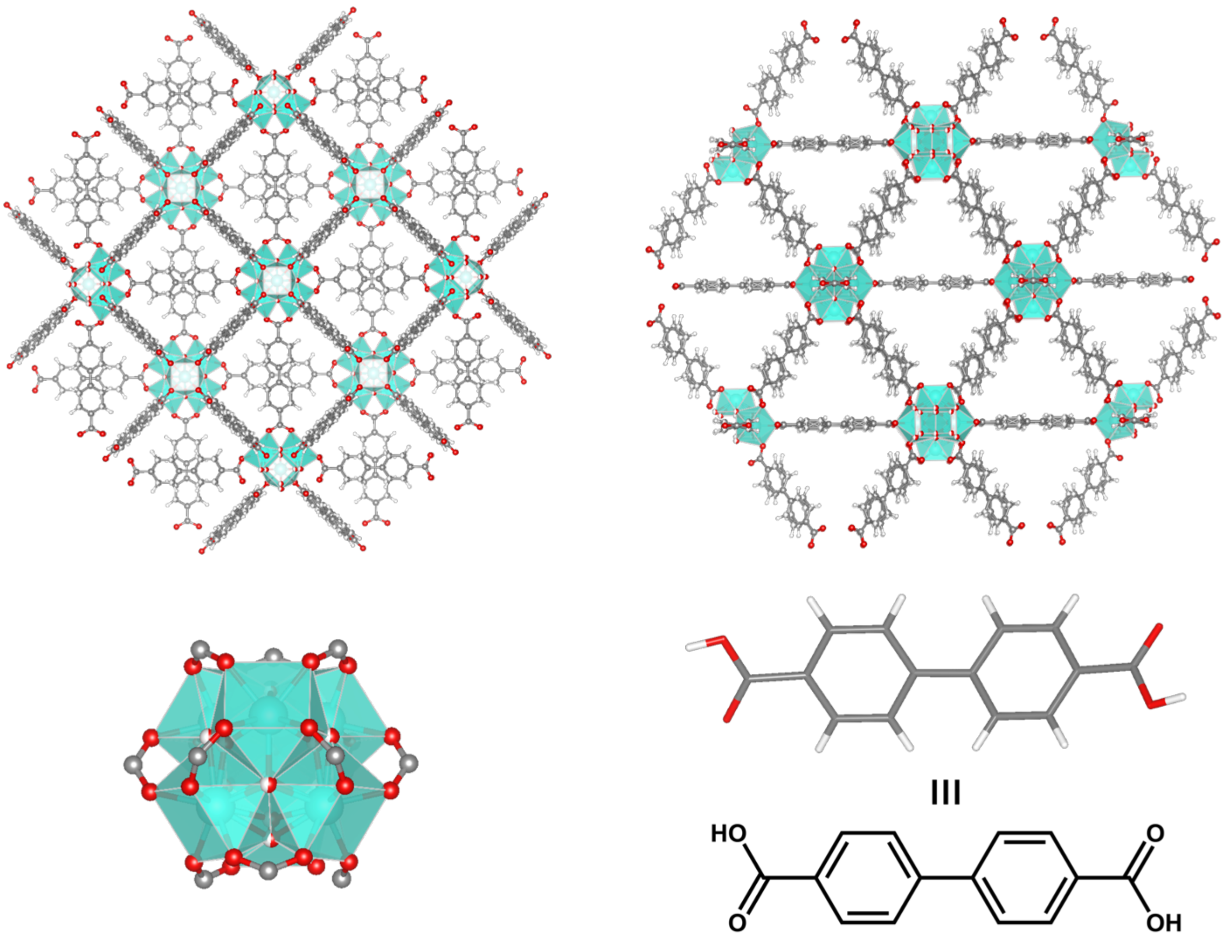


**Supplementary Figure 63**. (*top*) Two views of the single crystal X-ray structure of UiO-67.[18] (*bottom*) The metal node of UiO-67 and the structure of the organic linker, 4,4'-biphenyldicarboxylic acid ($H_2BPDC$), used for MOF preparation. The aqua polyhedra represent zirconium atoms, and the red, gray, and white spheres represent oxygen, carbon, and hydrogen atoms, respectively.

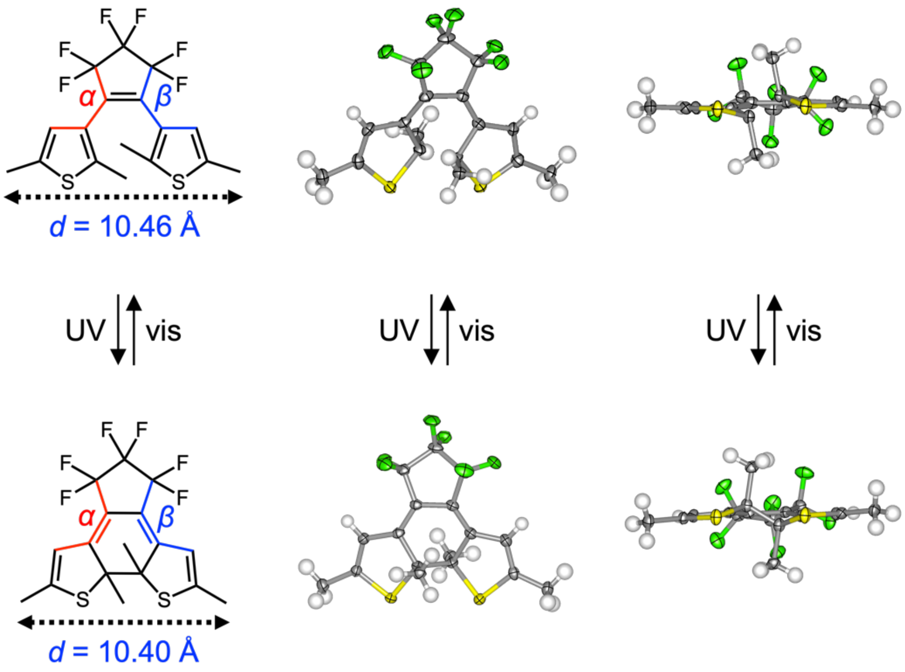


**Supplementary Figure 64**. Two views of the X-ray crystal structures of the open and closed isomers of a diarylethene derivative demonstrating the minimal structural rearrangement that occurs upon photoisomerization. Photoisomerization coincides with a change in the dihedral angles, *α* and *β*, of 0.5° and 0.4°, respectively, as well as a change in length of 0.06 Å. The gray, green, yellow, and white ellipsoids represent carbon, fluorine, sulfur, and hydrogen atoms, respectively. Thermal ellipsoids are drawn at the 50% probability level.[19]

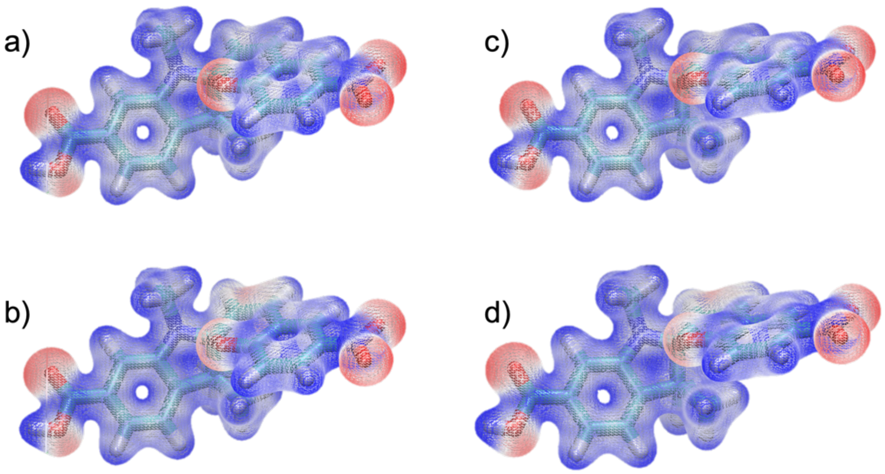


**Supplementary Figure 65**. Electrostatic charge distributions of non-integrated **2** in the ground and excited states (a and b, respectively), as well as coordinatively-integrated **2** in the ground and excited states (c and d, respectively). All electrostatic charge distributions were computed using TD-DFT at the B3LYP/6-31G* level. The molecules were visualized with VMD 1.9.4[12] at the density surface using an isovalue of 0.035, with the coloring of the electrostatic potential ranging from −0.08–0.20, all in atomic units.

**Supplementary References**.

1. Williams, D. E. *et al.* Flipping the Switch: Fast Photoisomerization in a Confined Environment. *J. Am. Chem. Soc.* **140**, 7611–7622 (2018).

2. Raghav, N. *et al.* In-vitro studies of various carbonyl derivatives on liver alkaline phosphatase. *J. Chem. Pharm. Res.* **2**, 801–807 (2010).

3. Thaggard, G. C. *et al.* Confinement-Driven Photophysics in Hydrazone-Based Hierarchical Materials. *Angew. Chem. Int. Ed.* **62**, e202211776 (2023).

4. Chaur, M. N., Collado, D. & Lehn, J.-M. Configurational and Constitutional Information Storage: Multiple Dynamics in Systems Based on Pyridyl and Acyl Hydrazones. *Chem. Eur. J.* **17**, 248–258 (2011).

5. Zhang, M. *et al.* Hydrazone-Based AIEgens with Photofluorochromic Ability for Rewritable, Intensity-Variable, and High-Resolution Photopattern. *Adv. Funct. Mater.* **33**, 2213927 (2023).

6. Martin, C. R. *et al.* Stimuli-Modulated Metal Oxidation States in Photochromic MOFs. *J. Am. Chem. Soc.* **144**, 4457–4468 (2022).

7. Karmakar, M., Bag, S. K., Mondal, B. & Thakur, A. A conjugated photoresponsive dithienylethene–ferrocene system: applications in secret writing and decoding information. *J. Mater. Chem. C* **10**, 8860–8873 (2022).

8. Katz, M. J. *et al.* A facile synthesis of UiO-66, UiO-67 and their derivatives. *Chem. Commun.* **49**, 9449–9451 (2013).

9. Wu, Z. *et al.* Tetraphenylethene-Induced Free Volumes for the Isomerization of Spiropyran toward Multifunctional Materials in the Solid State. *ACS Appl. Mater. Interfaces* **10**, 30879–30886 (2018).

10. Park, K. C. *et al.* Capture Instead of Release: Defect-Modulated Radionuclide Leaching Kinetics in Metal–Organic Frameworks. *J. Am. Chem. Soc.* **144**, 16139–16149 (2022).

11. Bjorgaard, J. A., Kuzmenko, V., Velizhanin, K. A., & Tretiak, S. Solvent effects in time-dependent self-consistent field methods. I. Optical Response calculations, *J. Chem*. *Phys*. **142**, 044103 (2015).

12. Humphrey, W., Dalke, A., & Schulten, K. VMD-Visual Molecular Dynamics, *J. Molec*. *Graphics* **14**, 33–38 (1996).

13. Krause, L., Herbst-Irmer, R., Sheldrick, G. M. & Stalke, D. Comparison of silver and molybdenum microfocus X-ray sources for single-crystal structure determination. *J. Appl. Cryst.* **48**, 3–10 (2015).

14. APEX3 Version 2019.11-0 and SAINT+ Version 8.40B.

15. Sheldrick, G. M. SHELXT – Integrated space-group and crystal-structure determination. *Acta Cryst.* **A71**, 3–8 (2015).

16. Sheldrick, G. M. Crystal structure refinement with SHELXL. *Acta Cryst.* **C71**, 3–8 (2015).

17. Dolomanov, O. V., Bourhis, L. J., Gildea, R. J., Howard, J. A. K. & Puschmann, H. OLEX2: a complete structure solution, refinement and analysis program. *J. Appl. Cryst.* **42**, 339–341 (2009).

18. Ko, N. *et al.* A significant enhancement of water vapour uptake at low pressure by amine-functionalization of UiO-67. *Dalton Trans.* **44**, 2047–2051 (2014).

19. Yamada, T., Kobatake, S., Muto, K., & Irie, M. X-ray crystallographic study on single-crystalline photochromism of bis(2,5-dimethyl-3-thienyl)perfluorocyclopentene. *J. Am*. *Chem*. *Soc*. **122**, 1589–1592 (2000).
